# Mechanical, machinability and water absorption properties of novel kenaf fiber, glass fiber and graphene composites reinforced with epoxy

**DOI:** 10.1038/s41598-024-81314-0

**Published:** 2024-12-02

**Authors:** T. Narendiranath Babu, Shreyash Singh, D. Rama Prabha, Shubham Mishra, Vivek Pandey

**Affiliations:** 1 School of Mechanical Engineering, Vellore Institute of technology, Vellore, 632014 India; 2 School of Electrical Engineering, Vellore Institute of technology, Vellore, 632014 India

**Keywords:** Kenaf, Composites, Graphene, Alkali, Natural Fibre, Epoxy, Composites, Mechanical properties

## Abstract

This paper aims to fabricate a novel kenaf fiber, glass fiber, graphene composite reinforced with epoxy, and to study its water absorption, mechanical and machining properties. Natural fiber composites seek numerous applications nowadays due to its high strength is to weight ratio, high impact resistance, thermal stability, and recyclability. The literature study on natural fiber composites indicates lack of research on Kenaf fibers reinforced with E-glass fibers in conjunction with 0.5% and 1.0% of graphene. Hence, this research focuses on Kenaf fiber with percentage composition of graphene was varied as 0.5%, and 1.0%, by weight of the holding matrix and with/without incorporation of glass fiber. Suitable surface modifications were done by treating natural fibres by 0.5% NaOH for better adhesion of fibre and epoxy resin. Sonication and Cetyl trimethyl ammonium bromide (CTAB) treatments were also done to realize the fine scattering of graphene in the epoxy matrix in order to achieve better mechanical behaviour. Moulds were prepared as per D-638 ASTM standards. The treated fibres were then arranged in the mould by the conventional hand layup technique. The main objectives of this study are to conduct tensile, flexural, Impact, water absorption, machinability, and FTIR test. Result shows that the incorporation of graphene in natural fiber composites showed an increase in tensile strength, flexural strength, and water absorption properties by 27.7%, 53%, and 15% on average, respectively. The composites used in this research find their use in potential applications such as defence, biomedical aids, automobiles, packaging, actuators, etc. These often generate great interest, whenever there is a demand for fabricating lightweight and high strength material.

## Introduction

The main objective of this paper is to fabricate fiber-reinforced composites and compare their mechanical properties as well as water-absorbent properties. Overall interest and concern for the environment and human beings, the recent environmental regulations, and the unsustainable consumption of petroleum products have led to the rise and need for eco-friendly materials. The natural fiber is regarded as one of the eco-friendly materials with exceptional mechanical properties and an alternative to synthetic fiber. Natural fibers are defined as fibers that are neither synthetic nor human made. They are obtained from animals and plants^1^. The utility of natural fiber from both types of resources, renewable and non-renewable such as palm tree, sisal tree, flax, and jute to produce composite materials has gained immense attention in the past few decades.

NFPCs have high strength and specific stiffness. However, while discussing the limitations, natural fibers also have some inherent problems, and they have disadvantages in properties that are noteworthy. The chemical structure of natural fibers consists of lignin, hemicelluloses, pectin, cellulose, and waxy substances which allow absorption of moisture from the environment that causes weak and loose bindings between the polymer matrix and fiber. Furthermore, the adhesive and chemical bonding between polymer and natural fiber is considered a challenge due to different chemical structures of both matrix and fibers^2^. This causes ineffective transfer of stress load during the overlap of the finally produced composite material. Accordingly, modifications of natural fiber by using specific treatment processes are absolutely necessary. These modifications are usually performed by the use of chemical groups, which have the potential to react to the fiber and transform their chemical composition. This results in fiber modifications and helps to decrease the natural fibers’ moisture absorption rate, which causes good bonding between the fiber and polymer matrix^3^.

One of the significantly used and known natural fibers is kenaf fiber, which has been successfully utilized in a variety of applications. Kenaf (Hibiscus cannabinus L) is a summer season annual crop mainly grown for the fiber that is nearly related to jute and cotton crops. Since decades kenaf has been widely known as a cord-making crop to manufacture twine, rope, and sackcloth. Nowadays, there are numerous new applications for kenaf, which include building materials, paper products, absorbents, and cattle feeds.

At present, there is a rising interest in the utility of kenaf fibers, especially as composite reinforcement material. This is because kenaf possesses good mechanical behaviour and is able to grow quickly, rises to as high as 4–5 m within 4–5 months period growing season with the diameter of kenaf’s stalk up to 25–35 mm. It means that kenaf fiber composite is giving us the opportunity to manufacture products identical to those material properties of wood, but it takes the harvesting period of 150–170 days. Thus, it would massively decrease the demand for timber, which is now facing a major problem due to deforestation. Kenaf plant consists of a single and straight stalk that consists of a woody like core and fiber-like bark that surrounds the heart. Kenaf is comprised of 35–40% bast fiber and 60–65% core fibers by mass of the kenaf’s stalk.

The requirement for novel eco-friendly materials has resulted in the use of composites which is composed of natural fibres and polymer matrices, making it one of the most extensively researched areas in recent years. Experts have examined several fabrication methods utilized in the manufacturing of these composites and then offered a comprehensive assessment of the research focused on analyzing their structure and characteristics via various characterization approaches^4^.

The study’s domain is of significant importance to academia as well as industry, with global investigations into the use of cellulosic fibers in composite materials. Cellulosic fiber-reinforced composites have considerable strength, are low in weight, are nontoxic to human health and the natural environment, and are biodegradable, making them suitable for many applications. The topics discussed include techniques for treating fibers to improve the mechanical properties of composites; the processes involved in manufacturing; the effectiveness of hybrid composites; the impact of laminate arrangements; and the diverse applications of natural fiber composites^5^.

This survey presents a comprehensive analysis of various retting procedures, chemical and surface treatments, and characterization approaches for natural fibres. We summarize significant findings in the literature, emphasizing the treatment impacts on the characteristics of natural fibres^6^.

This study utilized graphene as a filler in epoxy composites reinforced with banyan aerial root fibers in different compositions. The combined influence of graphene and banyan fibres on the physical and mechanical properties of the epoxy thermoset was investigated. This article examines the optimal ratio of banyan fibres to graphene powder for the enhancement of epoxy composites. The ideal concentration of the composites was discovered to be four percentage graphene powder in the forty percentage banyan fiber-reinforced hybrid epoxy composites^7^.

Mixed coir-based natural fibre with different amounts of graphene powder (2.5, 5, and 7.5 wt%) and particles measuring 6, 18, and 25 µm are used in an epoxy matrix to see how the amount of nanofiller and the average particle size affected the improvement of mechanical properties. This study’s originality lies in its comprehensive analysis of the filler’s impact on particle size and its role in epoxy-based coir/graphene composites^8^.

The objective is to assess the hybridization impacts on various laminate stacking sequences composed of jute, kenaf, and e-glass textiles via the hoover bagging technique. The results suggest that the incorporation of glass textiles during hybridization could enhance the characteristics of jute/kenaf fabric-reinforced epoxy composites^9^.

This work aims to evaluate the hybridization impact of various laminate stacking sequences using jute, kenaf, and E-glass woven fabric by examining the physical and mechanical characteristics of nine distinct composites. The researchers produced the composite laminates using the hoover bagging technique. The testing of mechanical properties and looking at broken surfaces shows that jute/kenaf fabric-reinforced epoxy composites are much better at both tensile and flexural properties^10^.

Authors of this study have examined the room-to-low temperature (RT-LT) mechanical properties of liquid oxygen-compatible epoxy composites (LOC-EP) are crucial for the strength assessment of lightweight liquid oxygen tanks. This study initially examines the tensile and compressive mechanical properties of LOC-EP from room temperature to − 183 °C. The findings indicate that, in comparison to room temperature, the tensile strength, compressive strength, and elastic modulus at − 183 °C exhibit increases of 44%, 109%, and 160%, respectively. The proposed constitutive model is ultimately implemented in Abaqus via a user subroutine^11^.

The experts have identified the factors that make surface superhydrophilicity last and has come up with a way to make durable metallic surfaces that are both superwetting and superhydrophilic. The results make it clear how long-lasting superhydrophilicity works, which is helpful for real-world applications of surfaces that are both superwetting and superhydrophilic^12^.

Authors summarize the mechanical, and microstructural characteristics of nano-modified AACs. Nano-SiO_2_ was the predominant nanomaterial, representing 44% of the conducted research. According to the results, adding more nano-additives increases mechanical properties like being compressive, flexural, tensile, and impact strengths up to a positive point. However, these properties start to decline after that point^13^.

Experts examined the performance of epoxy composites reinforced with Prosopis juliflora fibre (PJF), treated with silane and containing Syzygium cumini filler (SCF), across many parameters. They did a lot of tests on the composites to see how the silane treatment and supercritical fluid (SCF) content changed their properties. These tests included thermogravimetric, Fourier transform infrared spectroscopy (FTIR), X-ray diffraction (XRD), 3D profilometry, and morphological ones. The mechanical characterization findings demonstrated notable enhancements in tensile strength, impact strength, and micro hardness in silane-treated composites with elevated SCF content relative to untreated samples and those with decreased SCF concentration^14^.

The research focused on the development and analysis of bio-based composites using moringa, bioresin, and hemp fibers as reinforcement. The composites were produced with four weighted percentage combinations of epoxy, moringa bioresin, and hemp fiber. We assessed the engineered composites based on their mechanical, thermal, water absorption, biodegradability, and morphological characteristics. The findings suggest that Moringa, bioresin, and hemp fiber composites may serve as sustainable substitutes for petroleum-based composites across several applications^15^.

Experts examined the creation of a product (helmet shell) by using thermosetting polymer waste (TPW) as a filler within a high-density polyethylene (HDPE) matrix. The HDPE and TPW were processed into extrudates via a twin-screw extruder. The mechanical characteristics were improved by the addition of up to 30 wt% of TPW. The results indicate that the suggested composite possesses adequate mechanical characteristics for the production of helmet shells^16^.

### Research gap

The literature study on natural fiber composites indicates a lack of research on Kenaf fibers reinforced with E-glass fibers in conjunction with 0.5% and 1.0% of graphene. Kenaf fibers possess a high strength-to-weight ratio, commendable tensile strength, and insulating qualities in comparison to synthetic fibers and metals. Amplifying this natural fiber composite with e-glass fibers, which possess more tensile strength than steel, can result in improved mechanical properties. This can offer a broad range of applications in aircraft, automotives, and several other sectors. Furthermore, prior investigations on kenaf fibres have not utilised various analysis such as tensile, flexural impact, water absorption and machinability. These approaches have the potential to reveal crucial details of the specimen, thereby clarifying the characteristics of the sample.

### Objectives

This research seeks to ascertain and thoroughly examine the tensile and flexural strengths of several Kenaf fibre composite variations. We concurrently apply and evaluate two distinct concentrations of 0.5% and 1% graphene to provide varying outcomes. We conduct this to gain insight and conclusive data about the graphene composition that produces the best output. We integrate and evaluate layers of E-glass fibers in precisely half of the produced specimens to assess the variation in the composite’s strengths. We construct the specimens, conduct tensile, bending, impact tests, water absorption and machinability test to study the characterization of the specimen. To study the chemical structure of composites and to examine the presence of various organic groups, facet binding between the fiber and the matrix FTIR was performed. A thorough literature review of kenaf fibre composites indicates that the fibre has been rigorously evaluated alongside numerous reinforcing elements, including graphene powder and polymer matrices. This work aims to evaluate the mechanical characteristics of kenaf fiber reinforced with e-glass along 0.5% and 1% of graphene, marking it as a pioneering study.

## Materials

### Kenaf fiber

Kenaf fiber has emerged as an essential plant, cultivated in developing and under-developed countries and has been regarded as an industrial crop^17^. Kenaf fiber can give mechanical properties that are almost near to the properties of synthetic fiber, which have a lower density than conventional materials, which results in light-weight and eco-friendly fiber-reinforced composites. The strength and performance of kenaf fiber-reinforced polymer composites are determined by various factors such as chemical treatment, the percentage of the kenaf fiber used, fiber content, and the shape of the fiber. The Kenaf fibres were used in this research. These fibres are procured from Fiber Region, Valasaravakkam, Chennai, Tamil Nadu. Density = 1.2 (g/cm^3^), Breaking Strength = 100.64(MPa), Elastic Modulus = 23(GPa), Tensile Strength = 283–800 (MPa).

### Glass fiber

Glass fiber is a material comprising of numerous extremely thin and fine fibers of glass. Furthermore, glass fibers are considered as versatile group of materials. These are used on a large scale as a reinforcement material for polymeric resins like unsaturated polyester and epoxy. Though glass fiber has the advantage of a very high strength property combined with low-density property at an affordable price, its stiffness is less than that of other used reinforcement fibers. Glass fiber will be continued to be utilized as one of the major reinforcement fiber in the future too.

### Epoxy LY556 & hardener HY951

It is a type of polymer or polyepoxide consisting of two or more epoxy groups. It is a thermosetting polymer that is produced by epoxy resin with a polyamine hardener. Araldite LY556 is the resin that is used in the production of composites. It is a non-modified liquid-epoxy resin. Its chemical structure resembles the epoxide family and is used for making the matrix phase. This epoxy resin is blended with the hardener HY 951 in a ratio of 10:1 by weight as prescribed.

### Graphene powder

Graphene is a popular allotrope of carbon that forms a single uniform layer of atoms that are arranged in a 2-D hexagonal lattice structure in which each of the vertexes is formed by one atom. It is also the basic element of other allotropes of carbon, for example, graphite, carbon nanotubes, fullerenes, and charcoal. Graphene possesses a unique set of properties that makes it different from other carbon allotropes. In comparison to its width, it is about many times stronger than the strongest steel. However, its density is immensely lower than steel, and it has a surface mass of about 0.765 mg per square meter.

The existence of more graphene leads to a better bonding in the composite matrix which ultimately results in an enhanced reinforcement effect. Previous literature have also proven that the functional groups present on the graphene interface increase the degree of cross-linking in the specimens which ultimately improves the tensile and flexural property of composites. One per cent of graphene is optimum with e-glass fibre and kenaf because of the synergistic effect. This effect takes place through the interaction of kenaf fibre and graphene.

The previous literature reported that the Incorporation of glass fiber in graphene reinforced natural fiber composites show increase in its mechanical strength. In the investigation, the result shows that the Kenaf-epoxy-Glass Fiber 1.0% graphene reinforced composite shows enhanced tensile and flexural strength. Therefore the combination of Kenaf Fiber + Epoxy + Glassfiber + 0.5% and 1% of Graphene were studied.

## Methodology

### Preparation of moulds and alkaline treatment of Kenaf fibre

#### Preparation of moulds

A mould is a hollow container that has a similar shape to the desired product. The mould acts as a medium in which the mixture is settled. The main purpose of the mould is to provide the desired shape to the mixture once it is dried. A mould is extracted from high-density fibreboard with a precise cut using CNC machine. The D638 ASTM standards are followed.

#### Alkaline treatment of kenaf fibre

Alkaline Treatment of Kenaf Fibre is a chemical treatment process, also known as mercerization, in which the fibers are submerged in an aqueous solution of NaOH (Sodium Hydroxide) with known concentration. The chemical treatment process alters and improves the surface of the natural fibers by pulling out and removing certain rates of hemicelluloses, lignin, wax, and various other substances, including oils that are wrapped over the outer surface of natural fibers. The mercerization has to be done carefully; if the treatment process parameters are not fulfilled, it can cause defibrillation in fibers. Further, it can also lead to pore formation and fiber embrittlement^18^.

$$Fiber - OH \, + \, NaOH \to Fiber - O - Na \, + \, H_{2} O$$

Alkaline treatment results in a rise in the overall density of natural fibers. It removes less dense non-cellulosic components such as lignin and hemicelluloses. After mercerization, the fibers gradually change their color and start appearing more yellowish. At the same time, when we keep on increasing the concentration rate of NaOH, the fibers start separating from one another while undergoing an alkaline treatment process, which increases the fibers’ active surface area. As a result, there is more surface area available on fibers for wetting and soaking by the resin. In addition to this, the crystallinity of the fibers, unit cell structure, and fiber orientation are also modified in the alkaline treatment process. Further, this chemical treatment process also improves the transfer of charge between the fibers and the matrix in the natural fiber composites and enhances mechanical properties^19^.

**Fig. 1:**
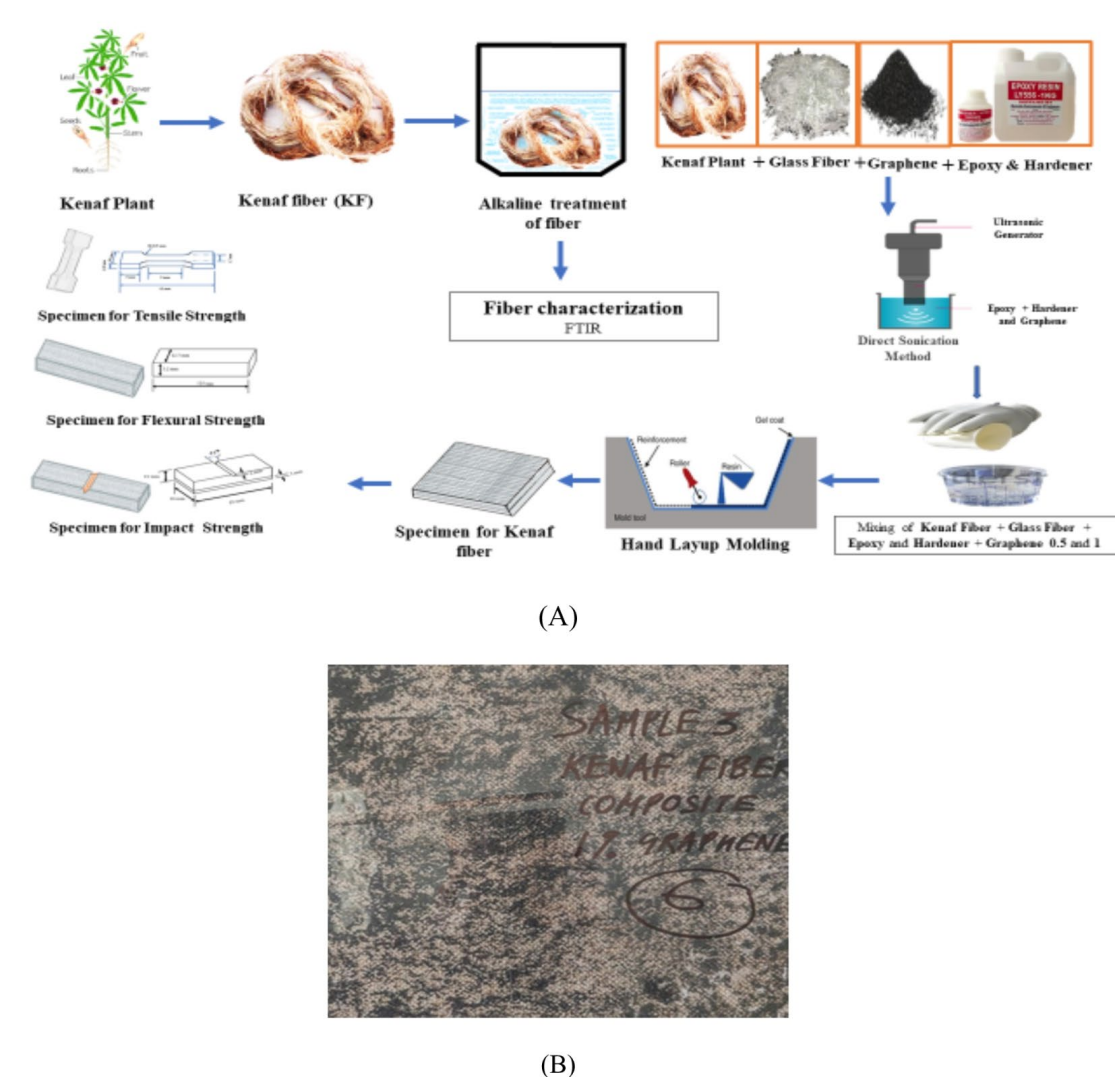
(**A**) Graphical abstract (**B**) composite panel prepared using an open mold process.

**Fig. 2:**
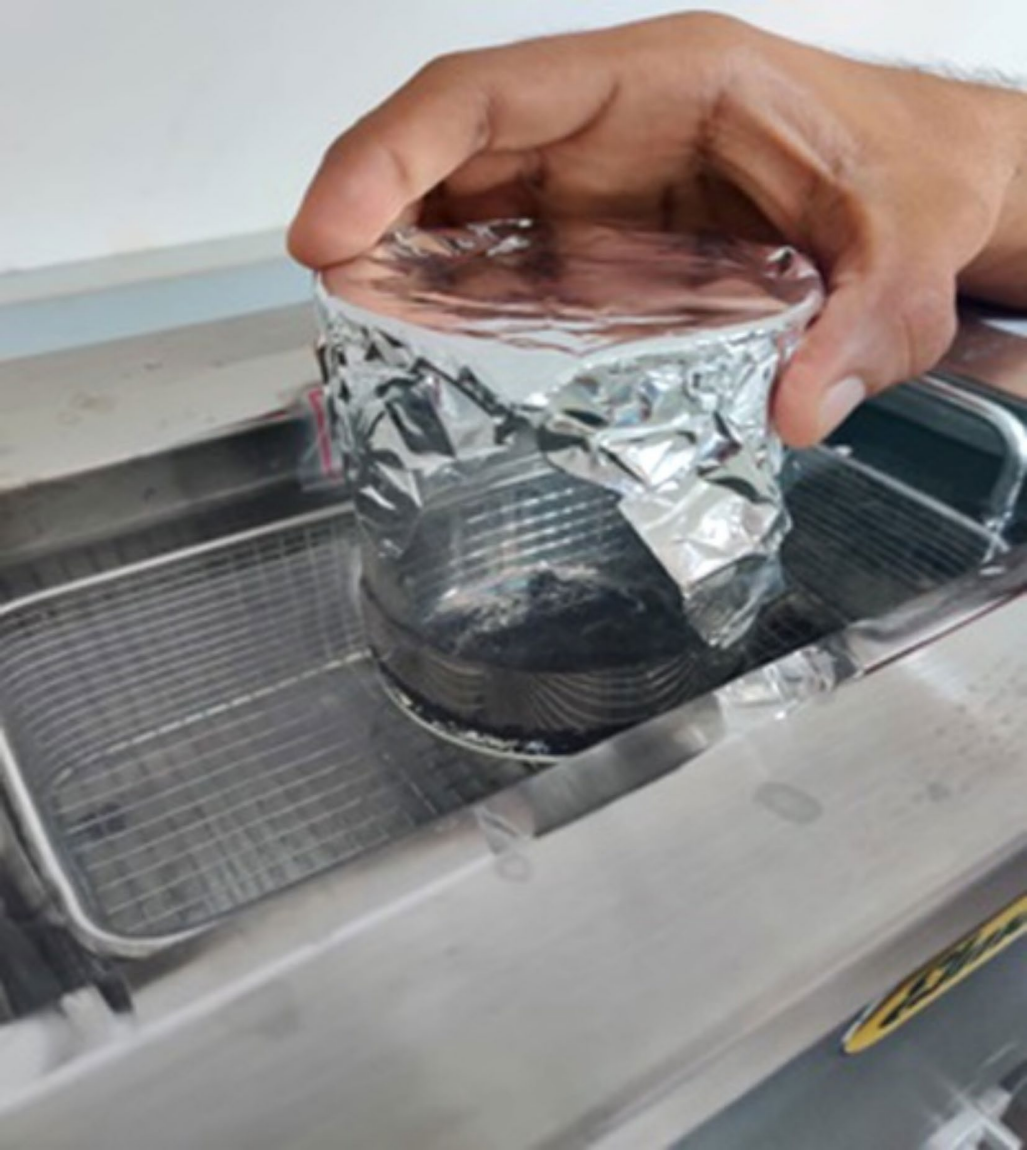
The Sonication process is done to agitate graphene particles in epoxy.

In the present experiment, the fibers are treated in one liter solution of 0.5%NaOH solution (Fig. 1). the natural fibers are kept in that alkaline solution for about 10 min. The process is done at room temperature (Fig. 2).

**Fig. 3:**
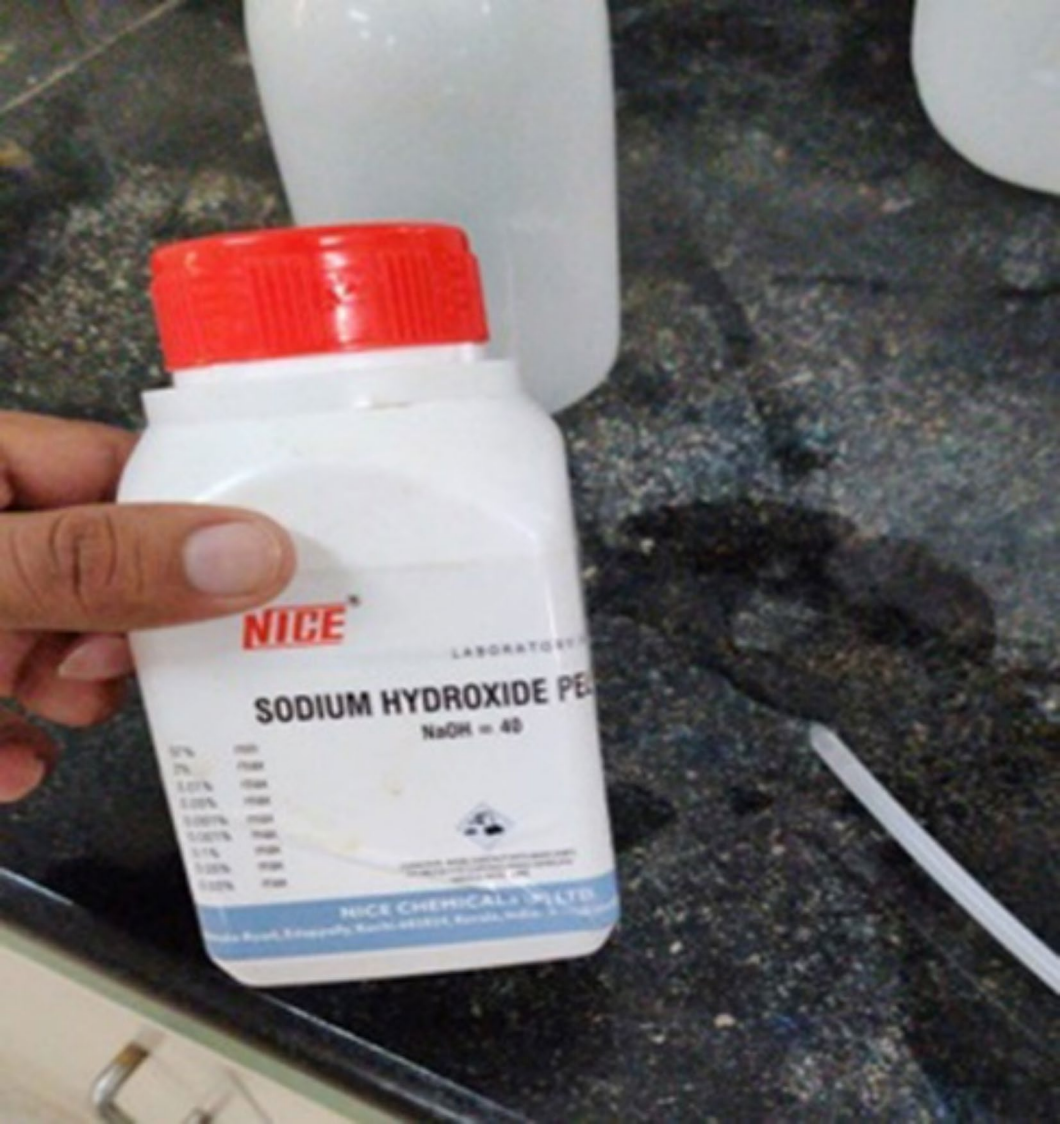
Alkali treatment of Kenaf fiber to enhance interfacial bonding.

Figure 3 shows alkali treatment of kenaf fiber to enhance interfacial bonding. After this process, the alkali treated natural fibers are washed and cleaned with distilled water at room temperature. Later on, the fibers need to be dried, which is done at room temperature at a humidity level of 70% for three days (72 h) to ensure adequate drying.

Then, the fibres are washed down with acetic acid to remove excess base out of it till pH value reaches seven on litmus paper. It can be tested using a litmus paper or a digital pH indicator. After acidic treatment, the fibres are washed with distilled water, at least three times.

After getting a neutral pH value of 0.3 percentage, the potassium permanganate solution is created to treat fibres, for half an hour. The fibres are allowed to dry at room temperature after which they are ready for sonication process and composite making process. Finally, the treated fibres are used for making composites after sonication process. Figure 1(a) shows graphical abstract.

### Sonication and incubation process

Sonication is the process of making fibers to agitate and to blend the particles in a liquid by applying the sound energy. This sound energy uses ultrasonic frequencies higher than 20 kHz, and so is also called as Ultrasonication. One can perform Sonication by applying two methods. It can be done either by using an ultrasonic bath or with the help of an ultrasonic probe colloquially, which is known as a sonicator.

In this process, the sound waves propagate into the aqueous medium, which leads to consecutive pressure difference cycle, i.e., low pressure (rarefaction) and high pressure (compression) depending upon frequency. Here the graphene used is Gpow P20 of particle size two μm and purity of 99.6% which was purchased from Tata Steel Plant, Jamshedpur, India. The graphene was dispersed in ethanol. Ethanol is a commonly used surfactant to disperse graphene. The process of dispersion of graphene in the solution of ethanol was carried out in a bath sonicator for 5 min (Fig. 2). Table 1 shows sample composition of composites.

**Table 1:** Sample composition of composites.

Sl. No	Sample code	Composition	Epoxy	Natural fiber	Glass fiber
			(%wt/wt)	(%wt/wt)	
1	A	Kenaf Fiber	75%	25%	-
2	B	Kenaf Fiber + 0.5% Graphene	75%	25%	-
3	C	Kenaf Fiber + 1.0% Graphene	75%	25%	-
4	D	Kenaf Fiber + Glass fiber + 0.5% Graphene	70%	20%	10%
5	E	Kenaf Fiber + Glass fiber + 1.0% Graphene	70%	20%	10%

The different weight compositions of graphene 0.5% and 1% were used to prepare a solution using sonication process. Then, the graphene powder was mixed with 100 ml methanol and CTAB one gram was then added based on weight percentage of graphene. The sonication was started after this mixture was created and continued for 2 h. During this development, water bath temperature level was sustained which did not surpass 25 °C to prevent evaporation. The 200 ml of epoxy was then mixed with sonicated mixture at 50 °C to evaporate methanol and constant stirring was done for proper mixing using the incubator machine.

Polyvinyl alcohol with five percentage of aqueous solution was used to coat multilayers on the surface of mould which helped to eject the specimens easily. Finally, the removal of specimen from mould took place, after drying of mould was complete which took twenty five days based on weather conditions.

## Results and discussion

### Tensile testing

Tensile testing was conducted on the samples carried out on Digital tensile testing machine TFT-10KN C ASTM D3039 specimens were prepared for testing purposes. A tensile force is applied at the rate of 2 mm/min. The gauge length of the specimen was kept at 100 mm. The Force v/s displacement graph is plotted until the sample fails. Ultimate tensile strength, Young’s Modulus of the material is known after conducting the tensile strength^20^.

#### Experimental procedure

**Fig. 4:**
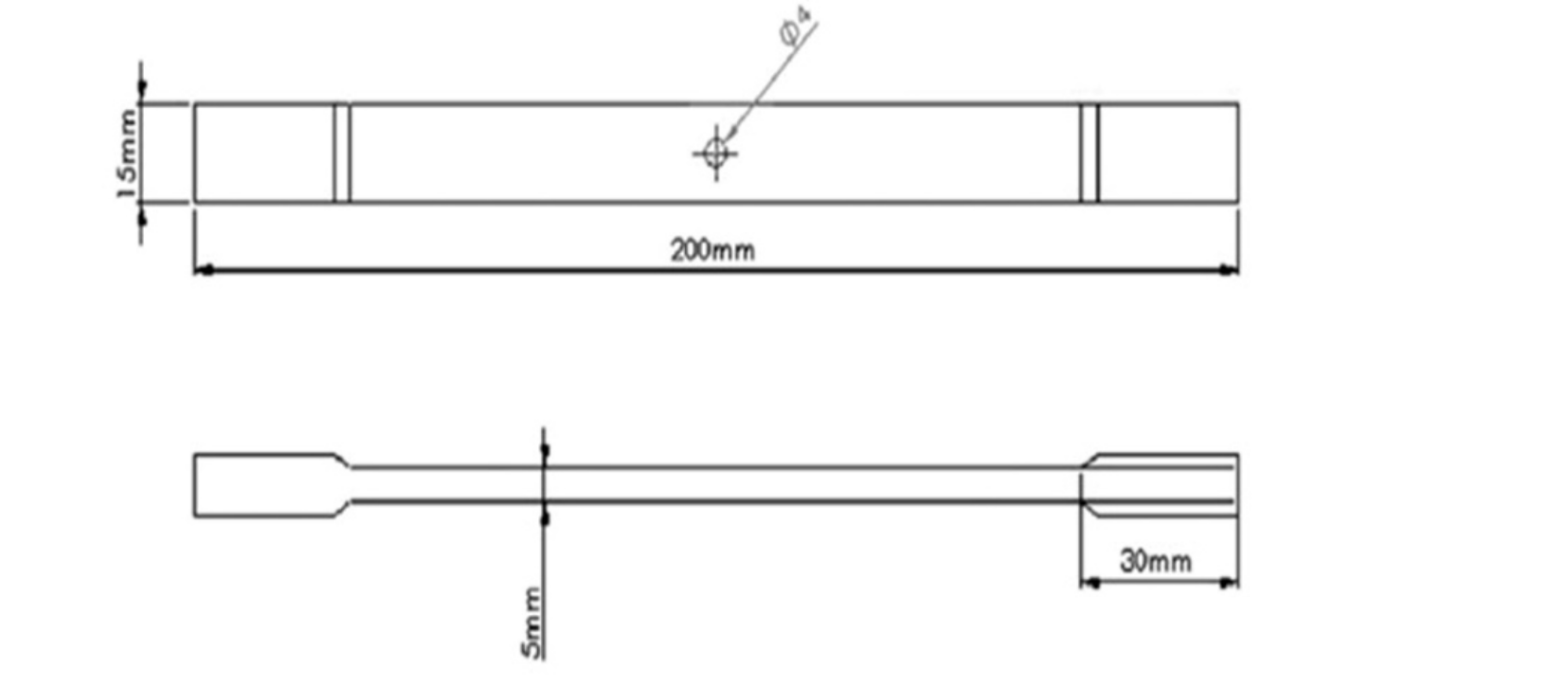
ATSM D3039 Standard for tensile testing.

- The specimen dimensions were checked as per **ASTM D3039** (Fig. 4) standard for easy clamping in the machine. The test samples are clamped in the machine.
- Then the feed required in the experiments was digitally entered in the computer, and the start button is clicked. The machine started gradually applying the load (tension) on the sample specimen.
- In this experiment, we moved the grips apart at the constant rate of 2 mm/min to stretch the specimen. The gage length of the ASTM D3039 standard specimen was 100 mm for 200 mm long specimen.
- The screen of the computer starts showing the graphs of stress v/s strain and another graph of force v/s displacement.
- On the other side, the ultimate force and ultimate tensile stress in a tabular manner was shown.
- The displacement and force on the sample specimen are continuously monitored, and the graph is plotted until the specimen fails.
- The specimen was cut into two pieces after the critical load applied to the specimen, as shown in the figure below. Table 2 shows results of tensile testing

**Table 2:** Results of tensile testing.

SNo	Sample	Composition	Ultimate force	Ultimate stress
1	A	Kenaf Fiber	1080 N	14.5 Mpa
2	B	Kenaf Fiber + 0.5% Graphene	1240 N	16.5 Mpa
3	C	Kenaf Fiber + 1.0% Graphene	1310 N	17.5 Mpa
4	D	Kenaf Fiber + Glass fiber + 0.5% Graphene	1460 N	19.5 Mpa
5	E	Kenaf Fiber + Glass fiber + 1.0% Graphene	1520 N	20.3 Mpa

**Fig. 5:**
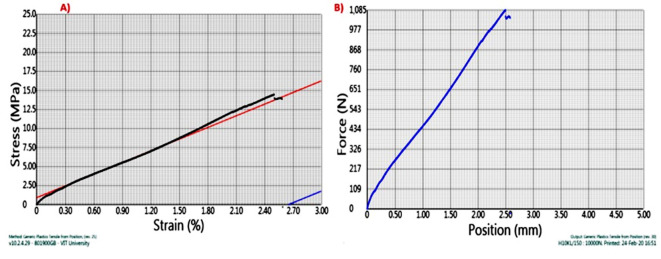
(**A**) Stress v/s Strain & (**B**) Force v/s displacement graph for sample A.

The Stress v/s Strain and force v/s Displacement graph was monitored for pure kenaf fiber composite as shown in Fig. 5. The results showed that Kenaf fiber composite showed an Ultimate tensile force of 1080 N, after which we can see that the specimen fails. It has Ultimate tensile stress of 14.5 Mpa, and a Young’ Modulus of 540 Mpa. The Stress–Strain Graph shows a linear line, and the displacement of the sample when a tensile load applied is approximately 2.50 mm.

**Fig. 6:**
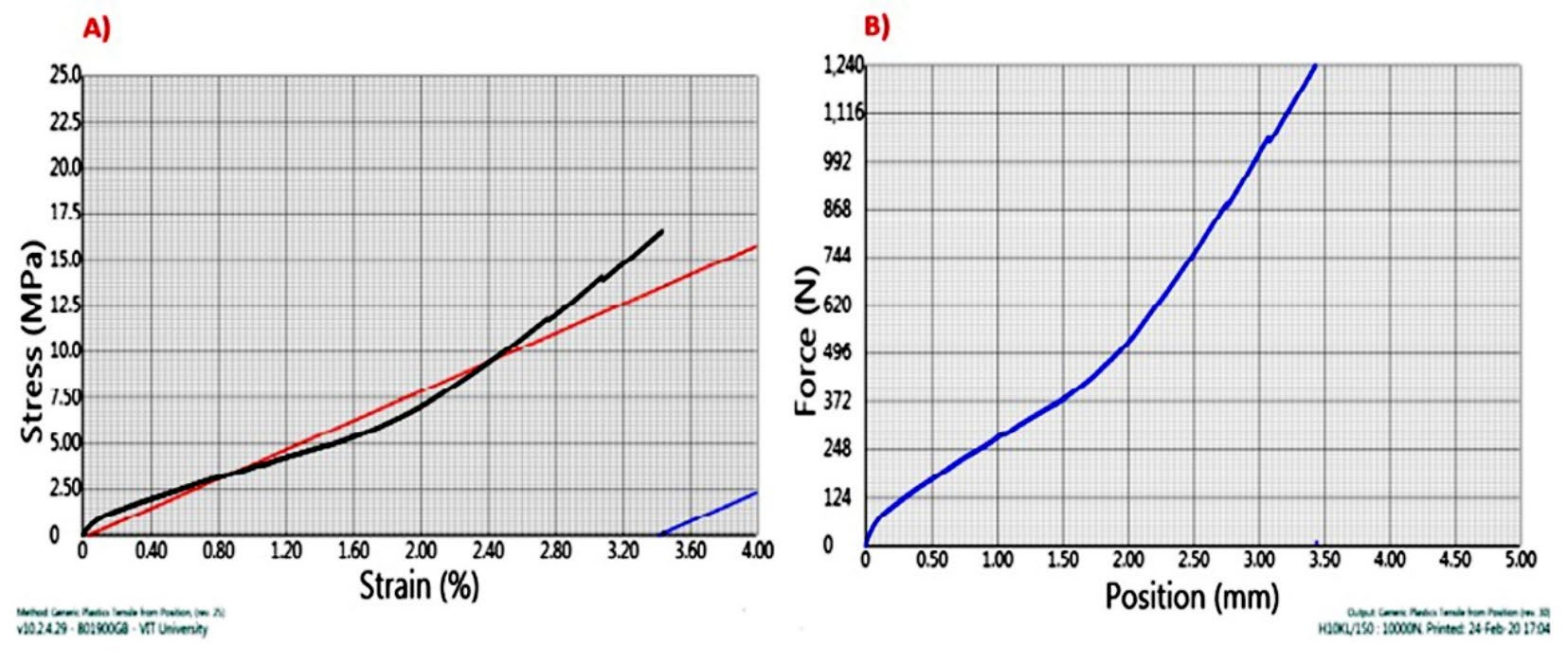
(**A**) Stress v/s Strain & (**B**) Force v/s displacement graph for sample B.

The Second sample as shown in Fig. 6, Kenaf fiber composite reinforced with 0.5% Graphene (by weight) showed an increase in the ultimate tensile strength, which can be referred as the inclusion of Graphene powder in the epoxy matrix increases the tensile strength of the composite. Graphene is reinforced in the epoxy matrix through the Sonication process, and it may be noted that graphene promotes the interfacial bonding between the fiber and matrix, which increases the strength. An addition of only 0.5% graphene (by weight) led to an increase in the Ultimate tensile force of the specimen by 60 N.

**Fig. 7:**
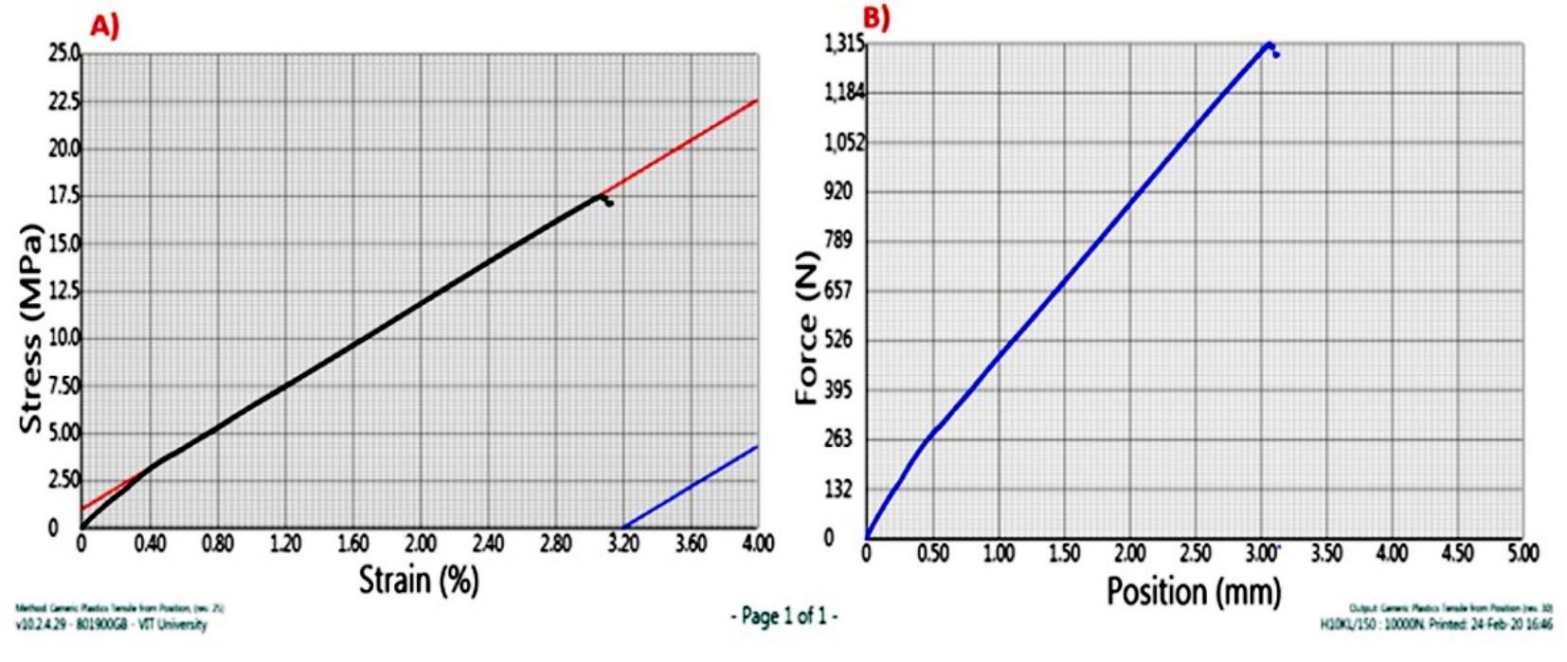
(**A**) Stress v/s Strain & (**B**) Force v/s displacement graph for sample C.

In the third sample as shown in Fig. 7. Further, an increase of 0.5% of graphene was seen in the composition of the composite. It was in literature reviews that with an increase in the content of graphene, the ultimate tensile force increases up to a specific limit and then gradually increases, if we keep expanding the graphene percentage in the composite, the ultimate tensile force will improve. Kenaf, fiber-1.0% Graphene, reinforced composites showed an ultimate tensile strength of 1310 N. Here; we can see that with only 2gm addition of graphene has led to the addition of 130 N of tensile force.

**Fig. 8:**
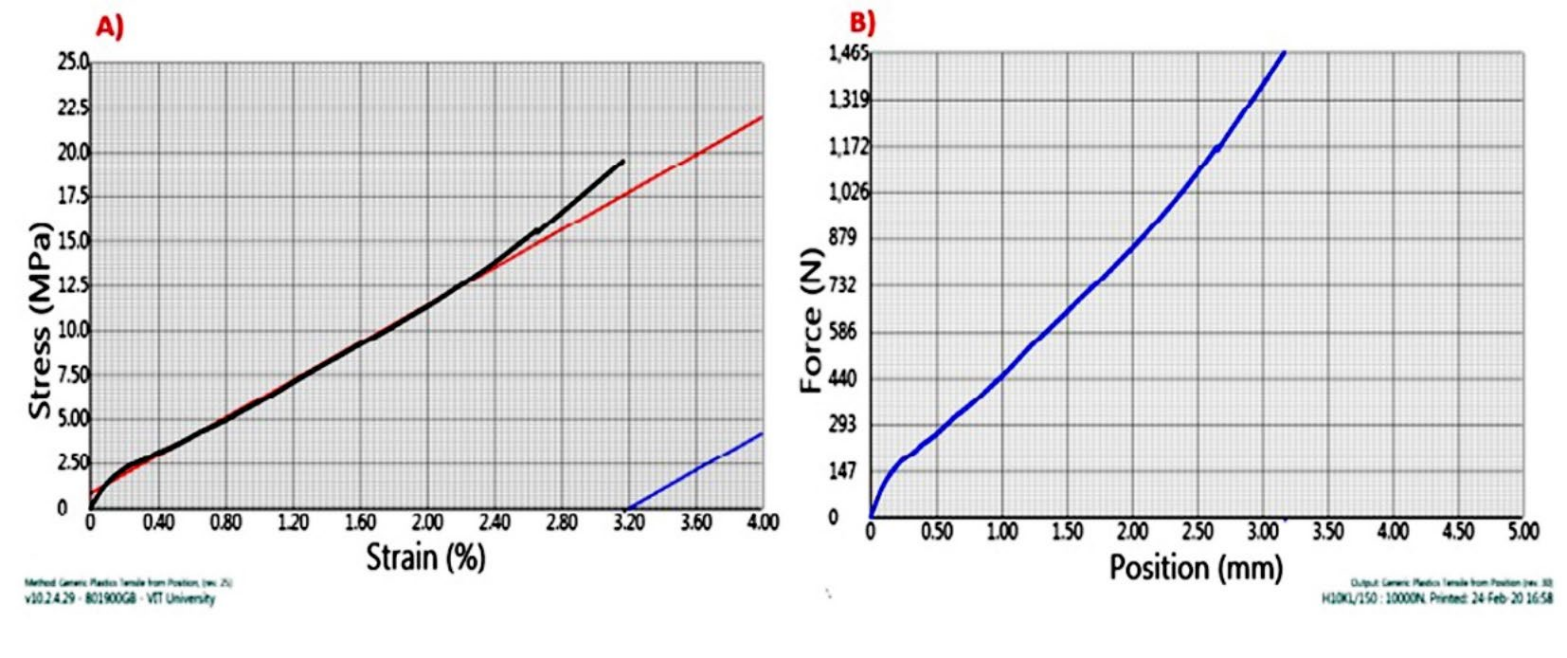
(**A**) Stress v/s Strain & (**B**) Force v/s displacement graph for sample D.

Sample D (as shown in Fig. 8) is a Kenaf fiber – Glass fiber – 0.5% Graphene reinforced composite. Here an addition of Glass fiber is seen. Glass fiber has good strength as well as excellent water retention properties. The UTS was found to be 1460 N, with an 11% increase in the tensile strength when compared to the latter. This proves that the incorporation of graphene and Glass fiber together shows a considerable change in the mechanical properties of the matrix.

**Fig. 9:**
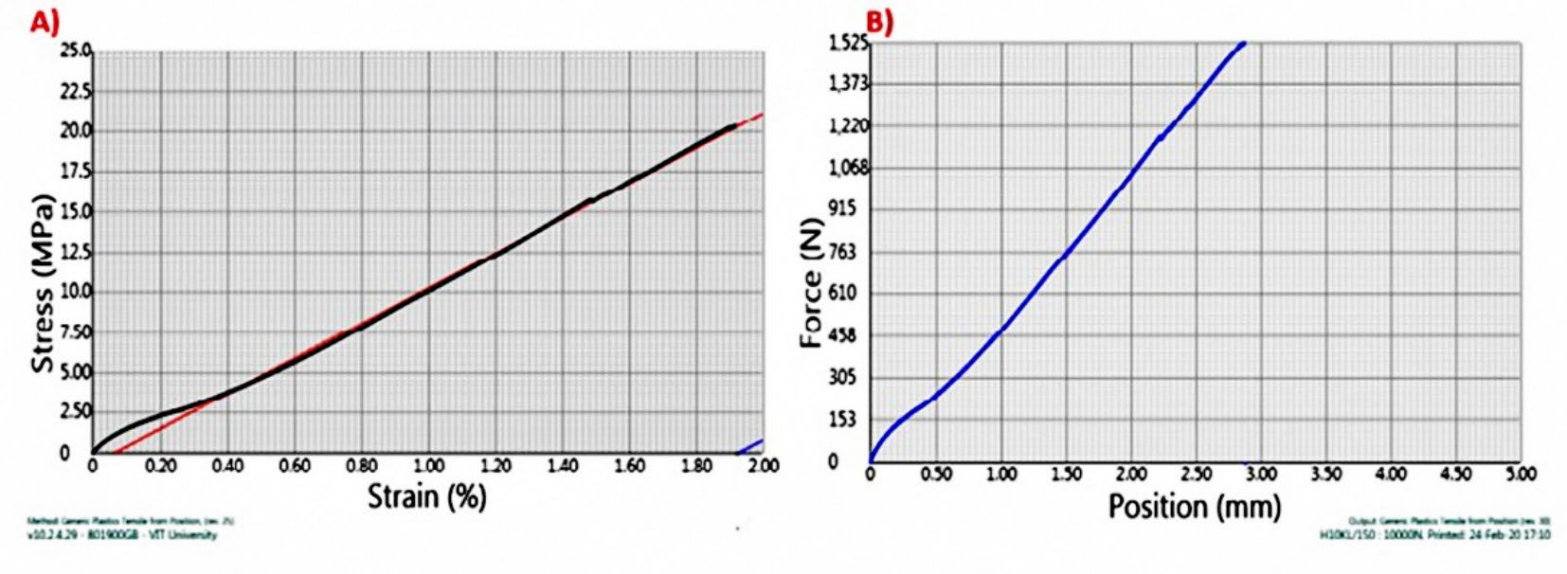
(**A**) Stress v/s Strain & (**B**) Force v/s displacement graph for sample E.

For the graphene reinforced kenaf fiber-Glass Fiber composites, tensile strength is most important. This is due to the better interface bonding of the fiber-matrix interface. This orientation tenders unvarying stress issuance in the composites and, therefore results in high tensile strength^21^. The variations in the force-position graph show that the modification in the tensile strength of the composites on glass fiber incorporation. It is from the Stress–strain graphs Sample A, Sample B, Sample C, Sample D, Sample E (as shown in Fig. 9) show an Ultimate Stress of 14.5 Mpa, 16.5 Mpa, 17.5 Mpa, 19.5 Mpa, 20.3 Mpa respectively. Graphene Reinforced (1% by weight)-Kenaf Fiber-Glass fiber composite show the highest strength.

#### Comparison on the basis of incorporation of glass fiber in Natural Fiber composites

E-glass incorporated fiber composites show better tensile strength than plain fiber composites^22^. The Ratio of Fiber: Epoxy in the Matrix is 25:75 (by Weight %). From the graph, it is evident that maximum load values for Kenaf-epoxy-Glass Fiber 1.0% graphene reinforced composite is 1520 N and for Kenaf-epoxy 1.0% graphene reinforced composite is 1310 N, respectively. Hence the former has higher tensile strength. The better adhesion in the matrix leads to a better reinforcement effect. More is the amount of epoxy; better is the adhesion effect. This leads to a desirable distribution and allocation of the natural fibers in the matrix, and the voids between the fibers are also bridged effectively. The stress distribution is favorable because of the presence of glass fiber, which results in better strength.

Based on graphene composition, another interesting result obtained was the Ultimate stress (US) of the composites. This US parameter is used to measure the ductility of the specimen up to fracture. The US values of 0.5% and 1% graphene composites were found to be 19.5 and 20.3 MPa respectively. US increases as the graphene composition increases. However, 1% graphene composite displayed exceptionally more US value. At the point when the maximum tensile load was applied on the composites, 0.5% graphene composite showed the minimum US whereas 1% graphene showed the maximum US. The results indicate that 1% is the ideal percentage composition which can withhold the maximum tensile load. Tensile modulus defines the stiffness of the composites which describes how much a composite can resist the tensile elongation when it is subjected to stress. The result shows that tensile modulus values have a direct relation to tensile strength. Therefore, more the strength of the composite then more is its ability to resist the tensile deformation.

### Flexural testing

**Fig. 10:**
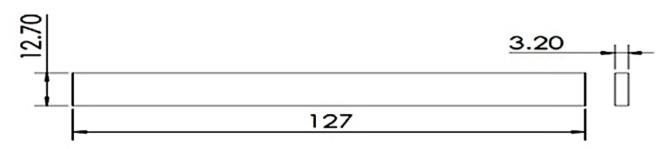
ASTM D790 standards for flexural testing.

The three-point bending test or flexural test was done on UTM using INSTRON 8801, as shown in the figure. Experiments were set up as per the ASTM D790 standard (Fig. 10). Experiments were performed at an average laboratory temperature of 30 °C and 65% RH. Gauge length was kept 100 mm for 125 mm long specimen. The force was concentrated at the center of the span through a loading cell for each specimen, and the strain rate or loading rate was 2 mm min^-1^. Force v/s displacement was continuously monitored using Blue hill version 5 software until the sample fails^23^. Table 3 shows results of flexural testing.

**Table 3:** Results of flexural testing.

SNo	Sample	Composition	Flexural force	Flexural extension
1	A	Kenaf Fiber	51.26 N	6.780 mm
2	B	Kenaf Fiber + 0.5% Graphene	62.62 N	5.157 mm
3	C	Kenaf Fiber + 1.0% Graphene	96.67 N	6.839 mm
4	D	Kenaf Fiber + Glass fiber + 0.5% Graphene	74.85 N	15.274 mm
5	E	Kenaf Fiber + Glass fiber + 1.0% Graphene	114.24 N	6.638 mm

The flexural test is important to know about the bending properties of the material and the composites. The researchers have designed this experiment very carefully by first experimenting with the pure fiber composite and then gradually adding graphene by 0.5%. The researchers have also added glass fiber to check the adhesive - compatibility of the glass fiber with natural fibers.

**Fig. 11:**
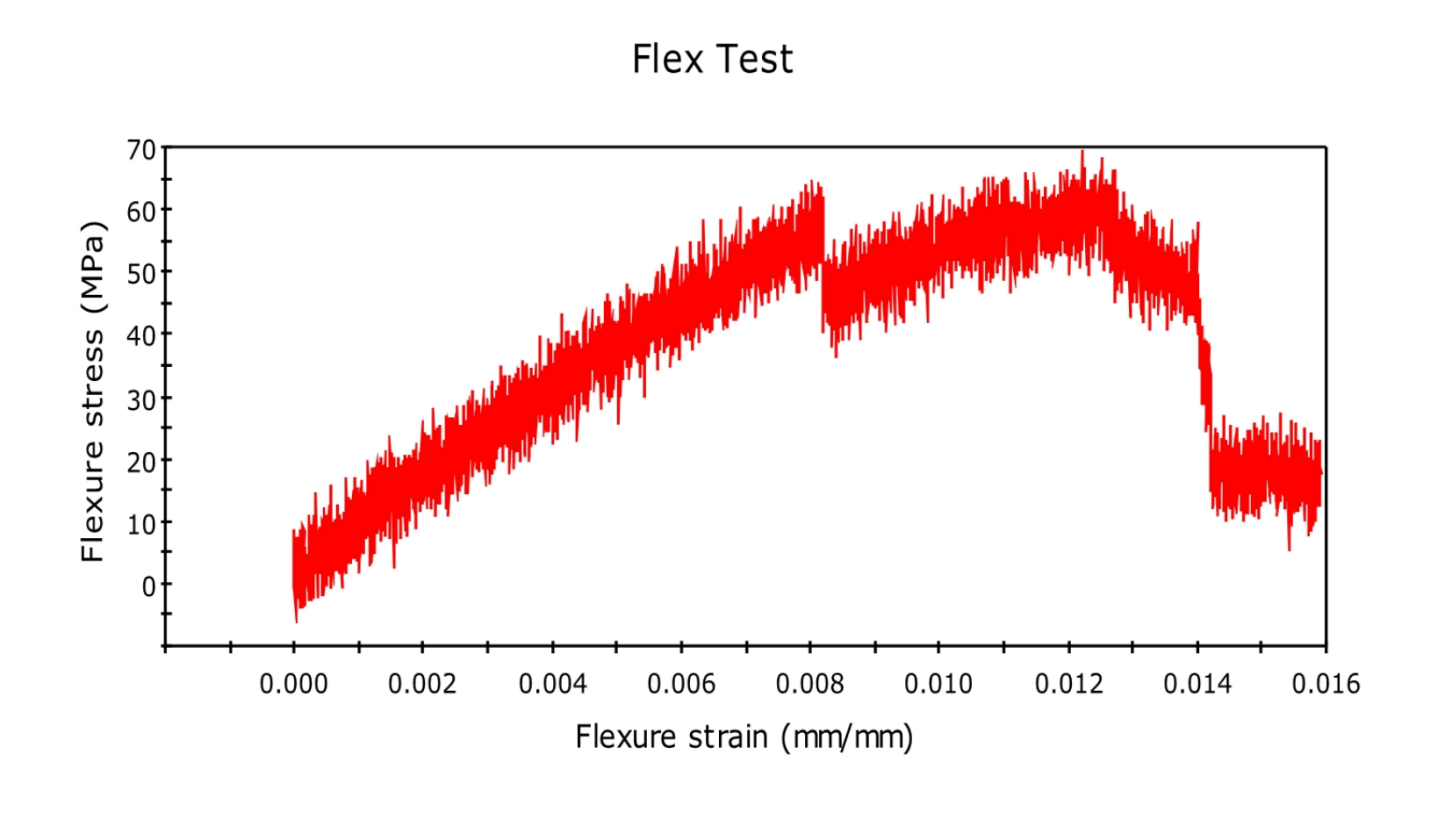
Stress-Strain Graph for Kenaf Fiber-Epoxy composite (A).

**Fig. 12:**
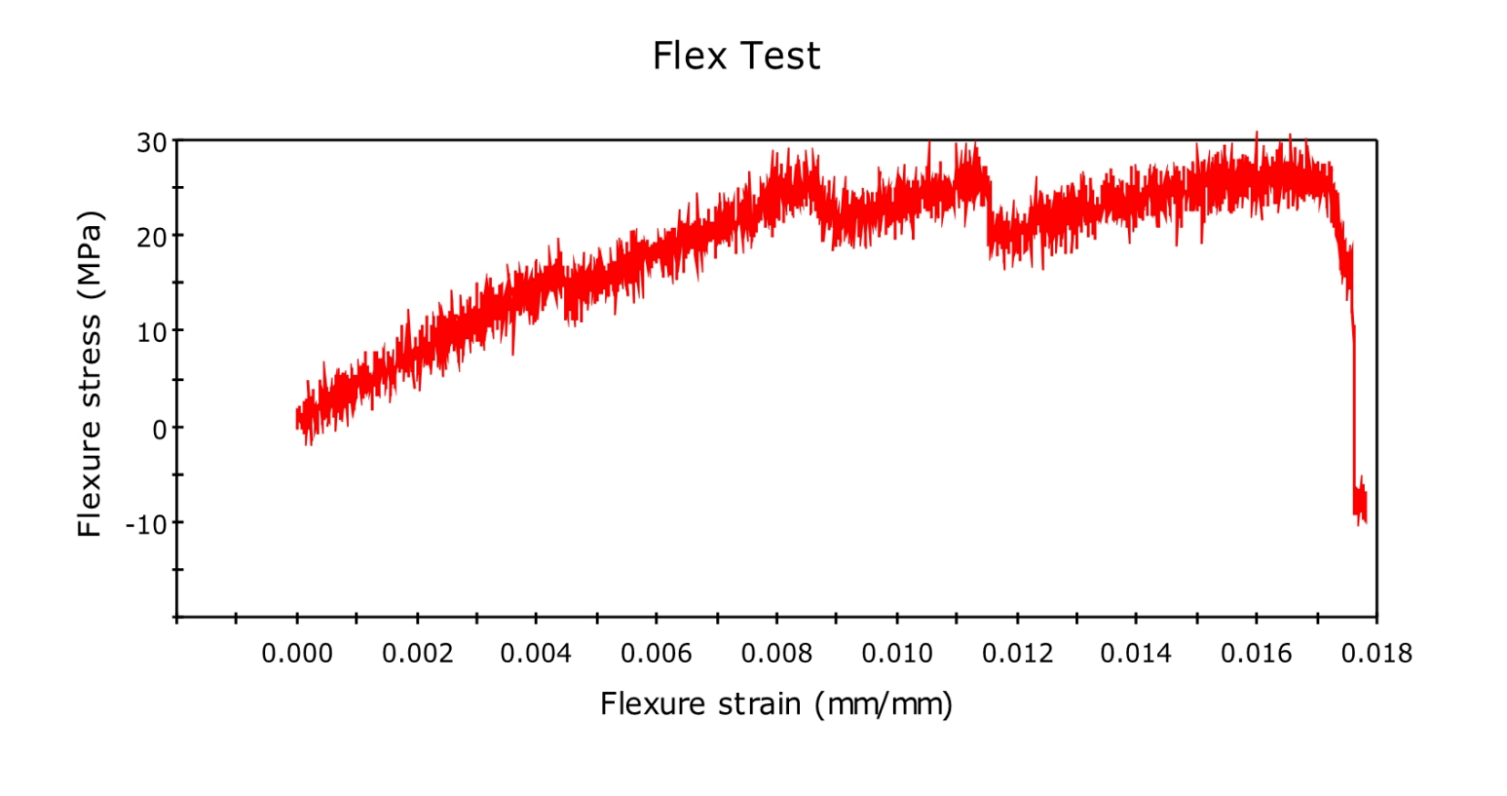
Stress-Strain Graph for Kenaf Fiber-Epoxy-0.5%Graphene Composite (B).

**Fig. 13:**
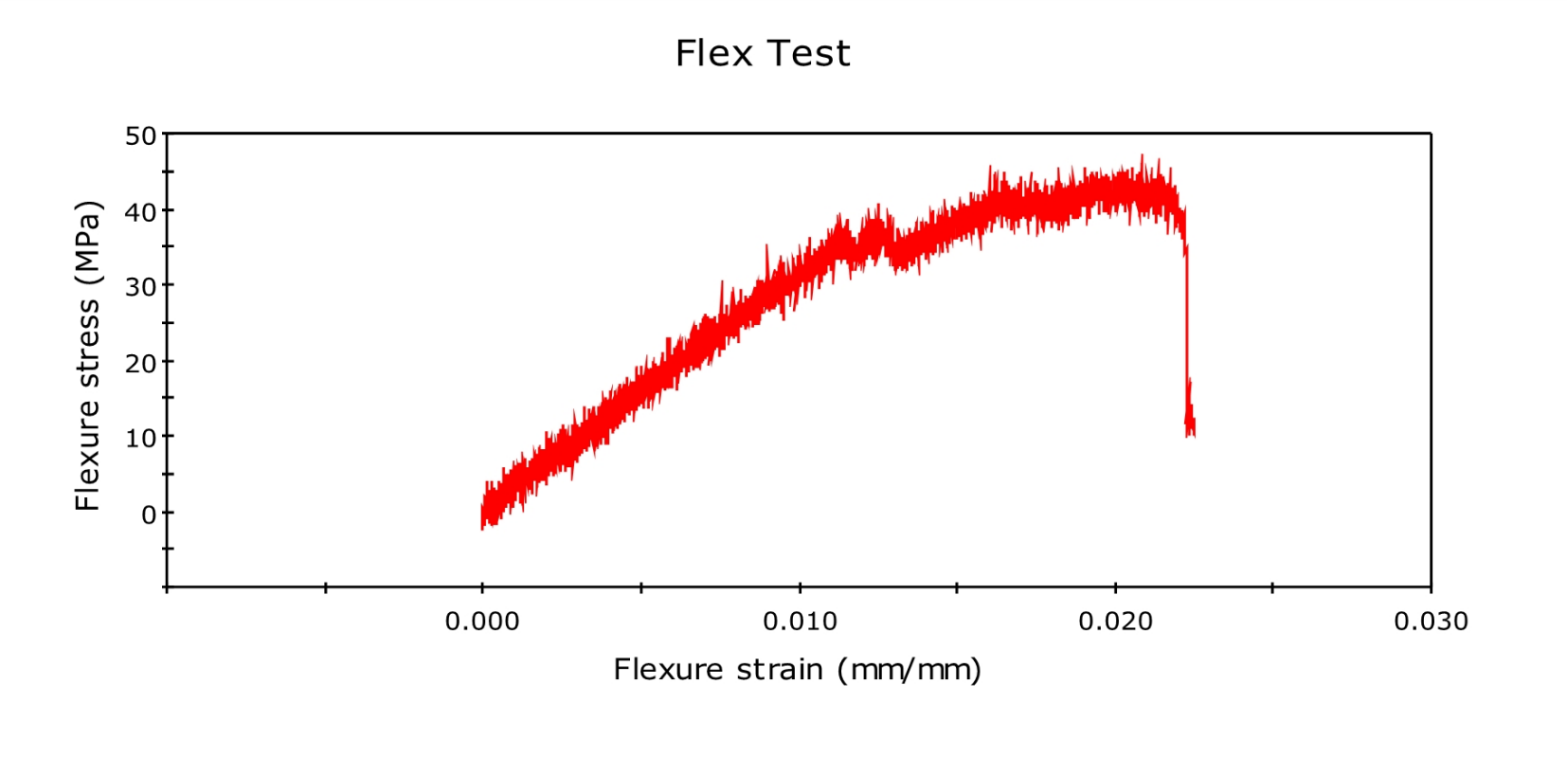
Stress-Strain Graphs for Kenaf Fiber-Epoxy-1.0%Graphene Composite (C).

Initially, we conducted the three-point bending test with pure kenaf fiber composite, and flexural force was found to be 51.26N, and flexural extension was found to be 6.780 mm. The related graph is shown in Fig. 11. flexural stress versus flexural strength. Now, we created new composites by adding 0.5% of graphene by weight. The corresponding value of force was found to be 62.62 N, while that of the flexural extension was 5.157 mm. This shows that the flexural strength of the natural fiber composites has increased by adding graphene into it. We further increased the graphene percentage to 1%, and the new values of flexural force were found to be 96.67N and that of flexural extension to be 6.839 mm. The relative values of flexural stress versus flexural strength curves are shown in Fig. 12 and Fig. 13 respectively. We observed a clear increasing trend of flexural strength when we increase the percentage of graphene.

In the second set of experiments, we added glass fiber 10% by weight with 0.5% by weight of graphene and performed a three-point bending test. The flexural force was found to be 74.85 N and while the flexural extension was found to be 15.274 mm. The flexural force showed a higher value than the composite having the same graphene percentage but no glass fiber.

**Fig. 14:**
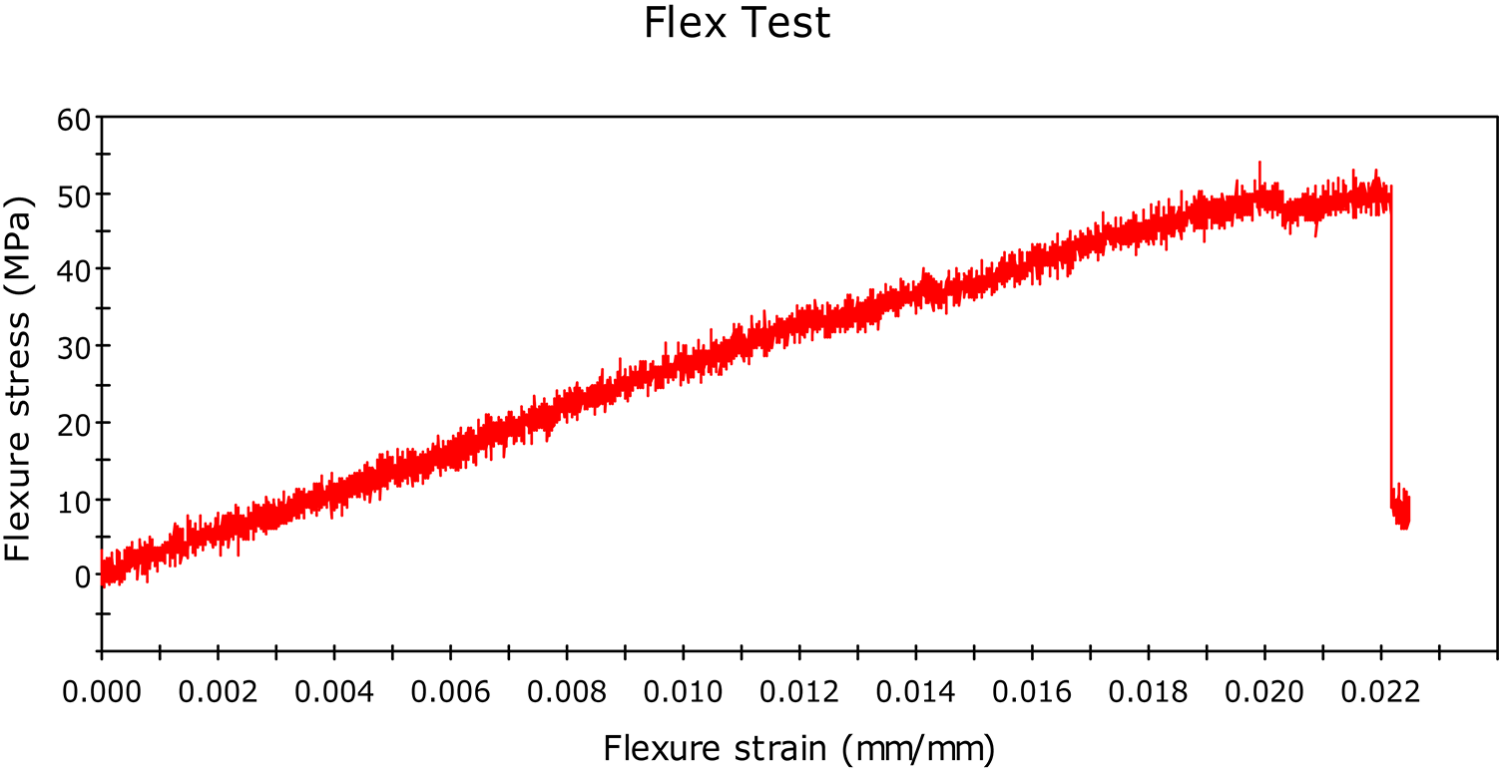
Stress-Strain Graphs for Kenaf Fiber-Epoxy—Gfiber-0.5%Graphene Composite (D).

**Fig. 15:**
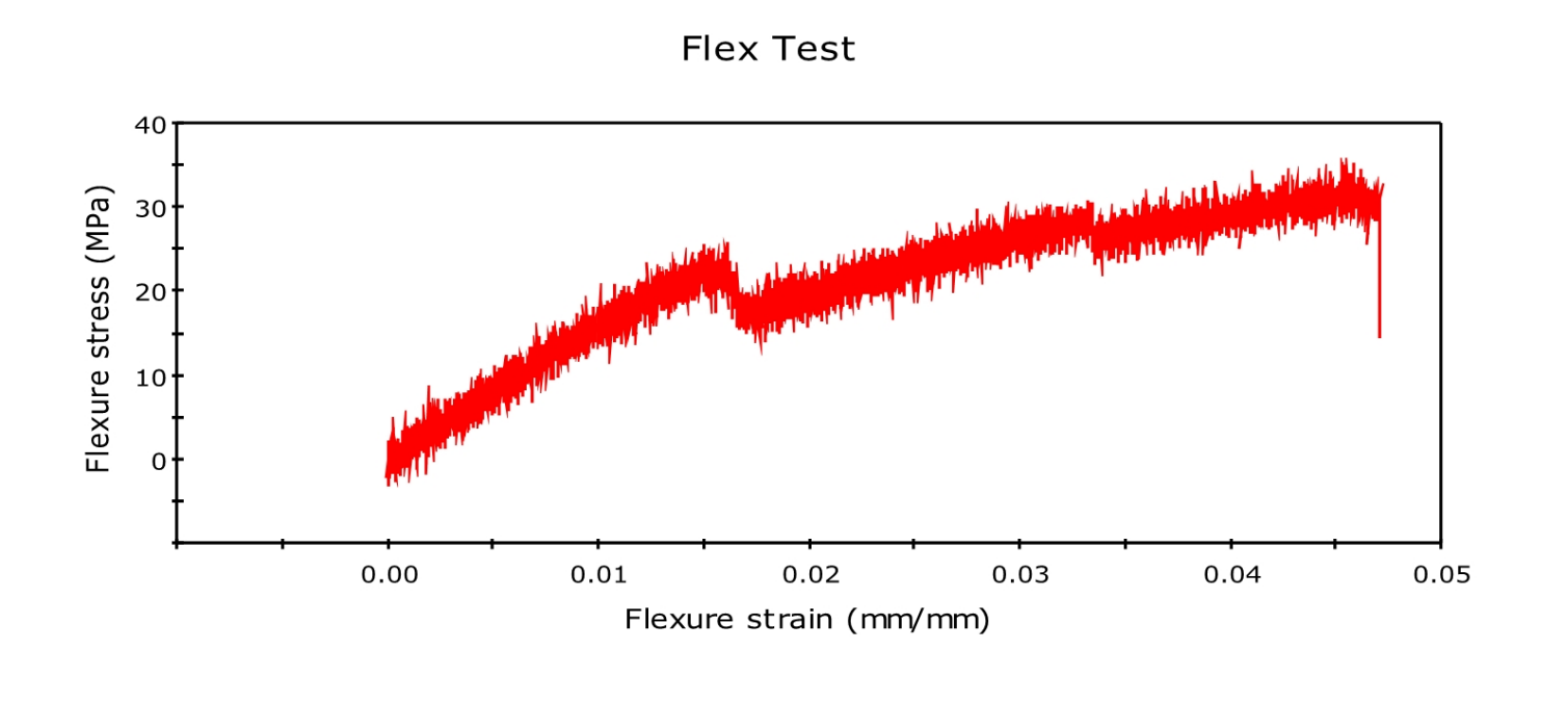
Stress-Strain Graphs for Kenaf Fiber-Epoxy-Gfiber-1.0%Graphene Composite (E).

We further performed the experiment by increasing the graphene percentage to 1%. The value of flexural force was found to be 114.24 N and flexural extension 6.638 mm. This again showed the increasing trend of flexural force by adding graphene. The related curve of flexural stress versus flexural strain is shown in Fig. 14 and Fig. 15.

The variations in the stress–strain relationship indicate the change in flexural strength of the composites on glass fiber incorporation and change in the composition of graphene in the holding matrix. From the results, it may be observed that Kenaf-epoxy-Glassfiber-1.0%. GKFC have preferred flexural quality on account of the fine structure of the kenaf and subsequently better support impact. Further, for each type of fiber composites, Glass Fiber incorporated composite with 1.0% graphene reinforced has the best flexural strength. Kenaf-epoxy-Glassfiber-1.0% GKFC has the most flexural strength value of 114 N.

The presence of more percentage of graphene leads to better adhesion bonding in the matrix which ultimately results in better reinforcement effect. Previous studies have also proved that graphene is known to increase the strength and stiffness of the composites. Synergistic effect is one of the main reasons why 1% graphene is optimum with kenaf and e-glass fibre. This takes place due to the interaction and bond created between e-glass fibre and graphene. Phenomenal characteristics shown by e-glass fibre is because of the crystallites structure in graphite interior which does contain graphene in interconnected manner, that too turbostratically arranged. Performance of the composites is majorly enhanced by the size and alignment in this structure. The graphite scaling of the size of crystallite eliminates the grain boundaries to an utmost extent in the lateral direction which brings the breakthrough and hence optimum results are obtained.

### Impact test

**Fig. 16:**
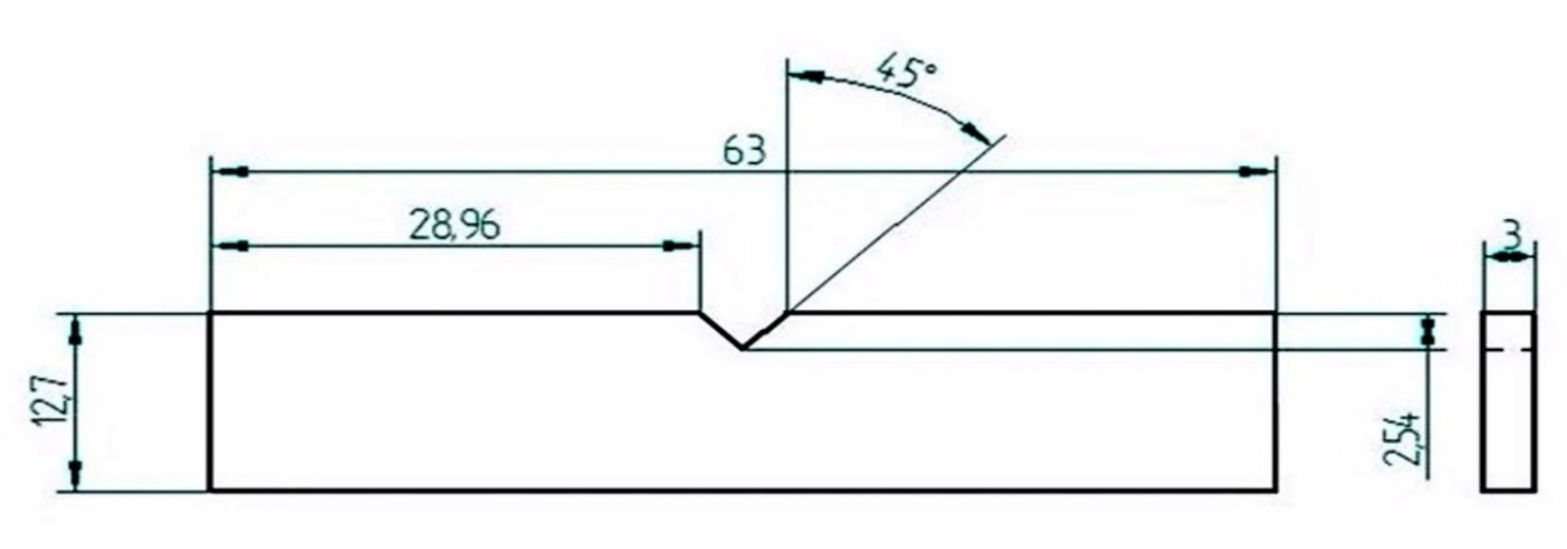
ASTM D256 Standard Specimen for Impact Testing.

The capacity of an object (Natural fiber composites) to hold out against High-rate loading is carried out in an Impact testing machine^24^. It determines the value of the amount of energy absorbed by the material during fracture on impact, which gives the value of its toughness. It is also used to differentiate and determine whether a material is brittle in nature or possesses more ductility. Usually, the service life or lifespan of a part or component is determined with the help of the Impact testing machine. Impact test or Charpy test was performed on Impact Testing Machine using FIT 300D, for the specimens made as per the ASTM D256 standard (Fig. 16).

The Impact Test was carried out on a Digital Impact machine, and ASTM D256 standard specimens were made for testing both Kenaf composites. Results were investigated, which showed that the incorporation of Graphene and Glass fiber results in the increase in the impact strength for Kenaf fiber composites. Table 4 shows results of impact testing.

**Table 4:** Results of impact testing.

Sl No	Sample code	Composition	Impact strength	Impact energy = impact strength/area
1	A	Kenaf Fiber	2.15 N	0.03 N/mm^2^
2	B	Kenaf Fiber + 0.5% Graphene	2.20 N	0.03 N/mm^2^
3	C	Kenaf Fiber + 1.0% Graphene	2.80 N	0.04 N/mm^2^
4	D	Kenaf Fiber + Glass fiber + 0.5% Graphene	3.20 N	0.05 N/mm^2^
5	E	Kenaf Fiber + Glass fiber + 1.0% Graphene	2.90 N	0.04 N/mm^2^

#### Experimental procedure

- V-notch; Depth = 2 mm; Angle = 45° Base radius = 0.25 mm
- A Charpy test sample specimen is placed along the parallel jaws symmetrically in the notch given in impact-testing machine. The impact measuring pointer is set up to its initial value.
- The Hammer is set-up to the initial position at the top.
- The Hammer is freed by releasing the breaker, and it goes from the initial height downward, hitting the sample.
- The specimen breaks as the Hammer hits it in the center.
- The value shown by the pointer and the energy absorbed are recorded.
- The observations and impact strength are tabulated as measured by the pointer.
- The above steps are repeated for another sample specimen.

**Fig. 17:**
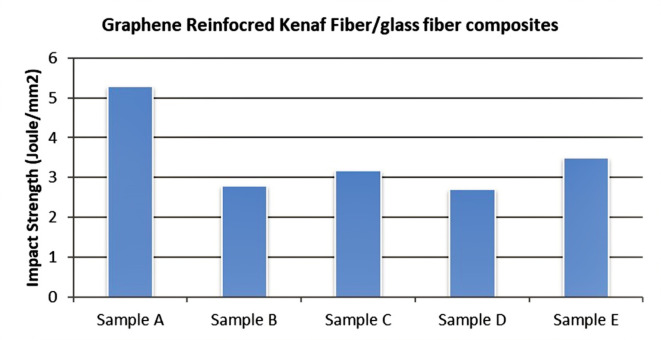
Graph for comparing Impact Strength of different samples.

From Fig. 17. is seen that the values of impact strength of Sample D i.e. kenaf fiber + Glass fiber + 0.5% Graphene is the highest. Incorporation of glass fiber in graphene reinforced natural fiber composites show increase in its impact strength which shows that these composites can withstand instant load.

### Water absorption test

A water absorption test is done on the specimens in order to find out the effects of humidity, rainfall and various other parameters on the natural fiber composites, in this experiment^25^. It determines the ability and rigidness of composites against these severe conditions. The water absorption test is done on our specimen according to ASTM standards. Table 5 shows results of water absorption test.

**Table 5:** Results of water absorption test.

SNo	Sample	Composition	Initial weight A	Final weight B	Water absorbed C = B-A	% Water absorbed C/A*100
			(M1)	(M2)	(M2-M1)	
1	A	Kenaf Fiber	1.7162	1.8068	.0906	5.28
2	B	Kenaf Fiber + 0.5% Graphene	1.9187	2.0156	.0969	5.05
3	C	Kenaf Fiber + 1.0% Graphene	1.8683	1.9576	.0893	4.78
4	D	Kenaf Fiber + Glass fiber + 0.5% Graphene	1.8704	1.9177	.4732	2.53
5	E	Kenaf Fiber + Glass fiber + 1.0% Graphene	2.6656	2.7242	.0586	2.20

*Weight of specimens’ is in grams.

#### Experimental procedure

- The specimen’s weight was taken in materials lab at VIT University, Vellore, India.
- It is done in an A&D GR-202 Semi-Micro Analytical Machine.
- Each specimen is weighted five times, and its mean value is noted.
- Weight of all the ten variety of specimens’ was noted in tabular form under Initial weight.
- Now the specimens were completely submerged in water at room temperature (20–30 °C) for one week to test its potential under harsh condition to find out its water absorption value.
- Then after 170 h (1 week), the same weighing process was repeated, and water absorption value is calculated for all the ten specimens’.
- It is to be noted that the lesser the water absorption value, the better is the composite.

$$W = \frac{M2 - M1}{{M1}} \times 100$$

M1 = mass of composite before performing the test, M2 = mass of composite after performing the test.

**Fig. 18:**
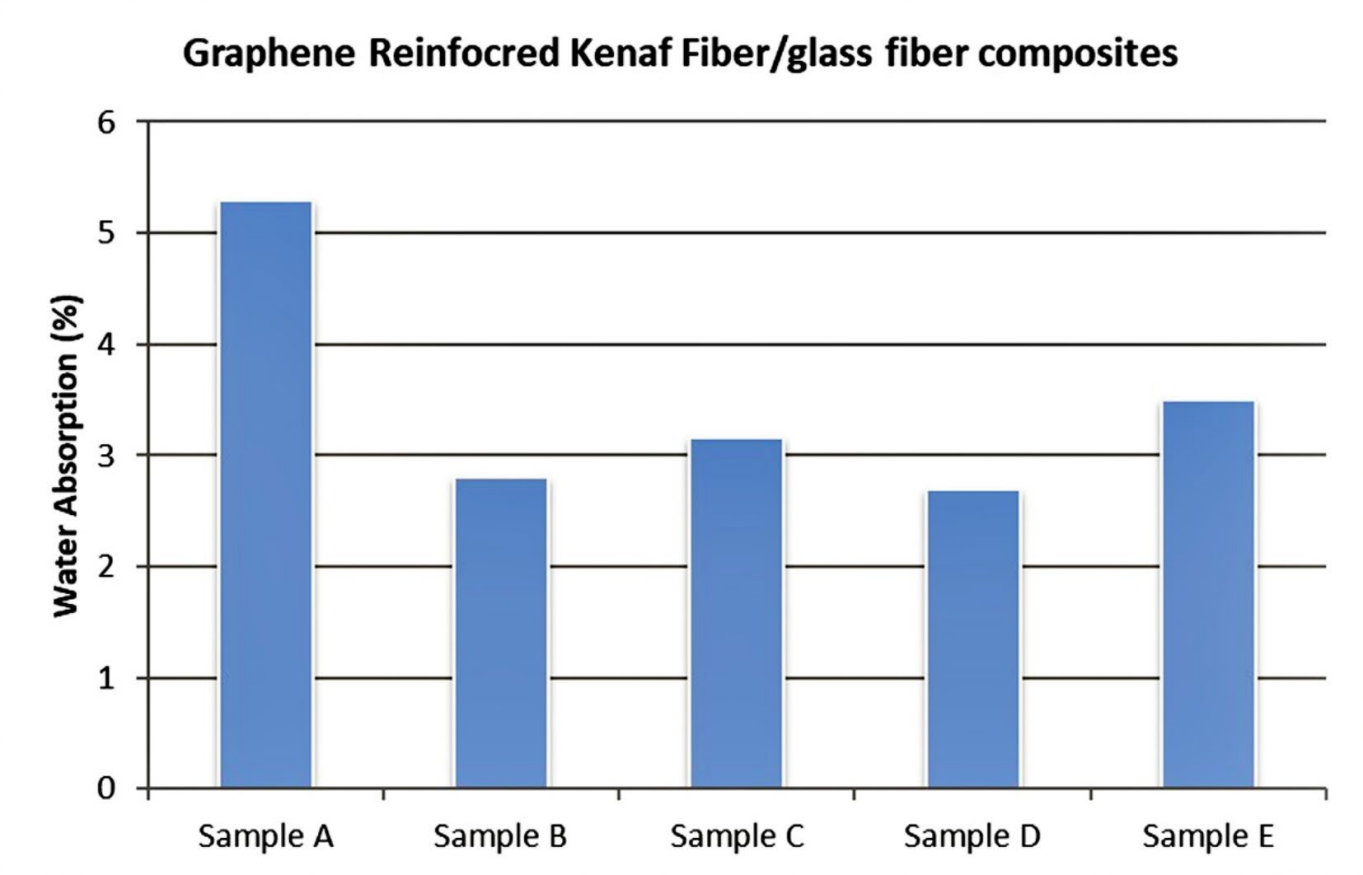
Graph shows water absorption properties (by %) of kenaf fiber composites.

The factors that influence water absorption tests are the type of specimen sample, temperature, and the materials used. After doing the test and critically analyzing the results obtained from our experiment with other research papers, we can conclude that our specimens’ consumed less water because of the occupancy of graphene particles in the matrix. It is observed that the specimen (Fig. 18) with a combination of graphene and a layer of glass Fiber gives the optimum result for kenaf fibers.

### Machininbality test

Drilling is one of the most important machining processes for composite materials because the parameter which is used for metal can’t be used for composite^26^. Hence optimum drilling parameters were obtained from design expert software. Table 6 shows DOE of Drilling. Design of experiment (DOE) was obtained for the speed ranges from 500 to 2000 rpm and feed ranges from 50 to 100 mm/min. Table 7 shows the optimum parameters for drilling and percentage of error. The Design of experiment (DOE) used while drilling is mentioned below.

**Table 6:** DOE of drilling.

Sl No	Speed (Rpm)	Feed (mm/min)
1	500	50
2	1000	300
3	500	300
4	1000	50
5	1100	500
6	1500	700
7	2000	1000
8	1100	1000
9	2000	500

**Table 7:** Optimum parameters for drilling and percentage of error.

Sample code	Speed (rpm)	Feed (mm/min)	Radius of hole drilled (mm)	Error %
A	500	50	2.840	5.3
B	500	50	2.916	2.8
C	500	50	2.905	3.167
D	1000	300	2.919	2.7
E	1000	300	2.895	3.5

*Sample D & E were drilled at a speed of 1000 rpm because of the incorporation of glass fiber.

The code which is used to operate CNC Milling Machine for drilling Purpose:

*M03 S _ EOB*

*G91 Z-7 F_ EOB*

*G91 Z* + *10 EOB*

*M05 EOB*

**Fig. 19:**
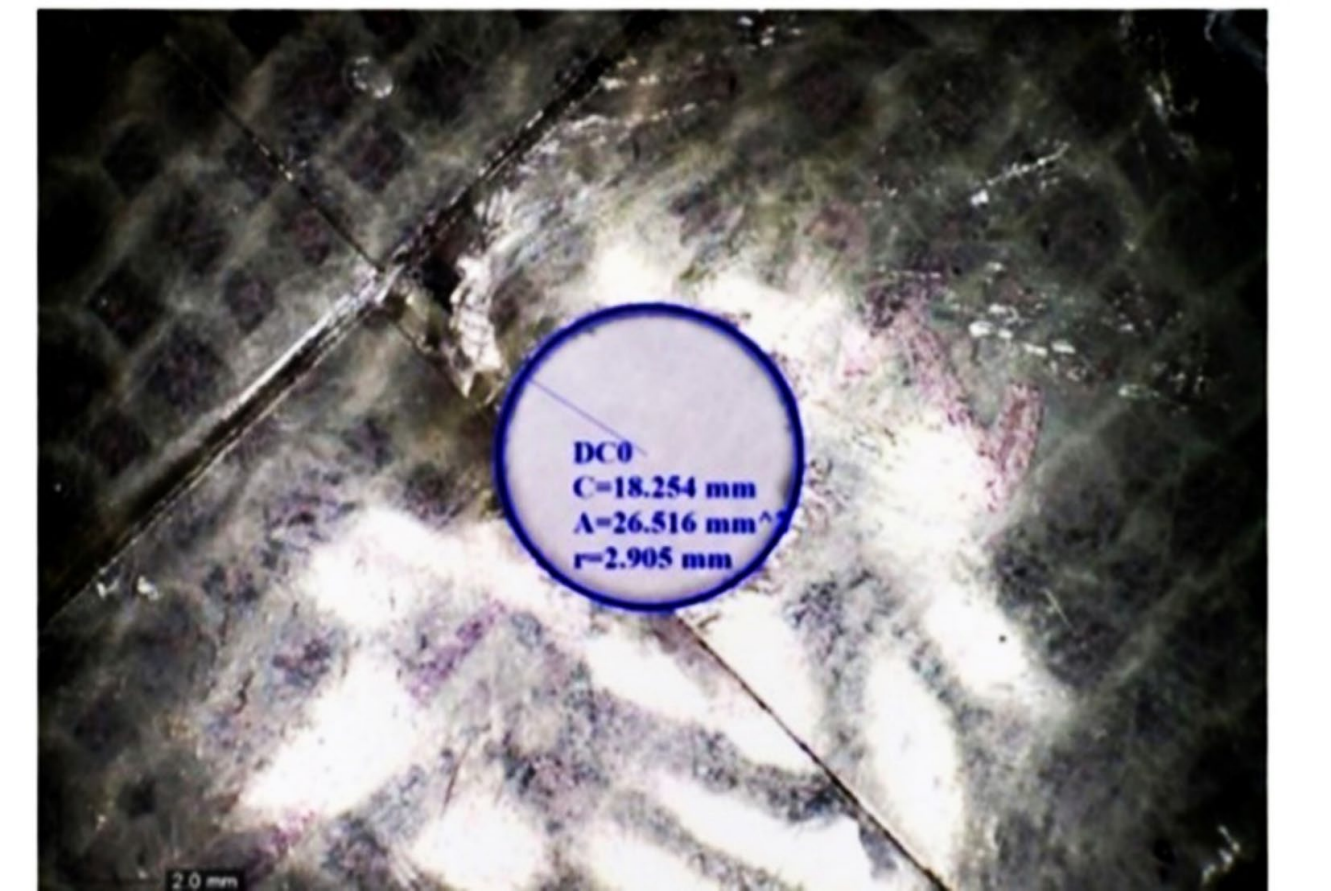
Ovality of Graphene reinforced kenaf fiber composite.

After S and F mention the Speed rate and corresponding Feed Rate, respectively, M03 is a command to start the milling operation, and M05 is to stop. Z-7 says that the milling should be done up to -7 in the Z direction from its initial position and Z + 10 to move up in + ve Z-direction up to 10 from its -7 position after milling is done as shows Fig. 19.

Nut and bolts are common instruments that are used to join the products. For this, the hole must be present on each of the surfaces. This makes drilling an important operation in manufacturing and automobile industries. Hence it becomes necessary to know how efficiently we can drill our materials to join the two objects. For drilling, a plat piece of dimension 80 mm X 85 mm was used from both the composites. To perform the operation, a drill bit of Ø3mm ± 0.1 mm was also used.

**Fig. 20:**
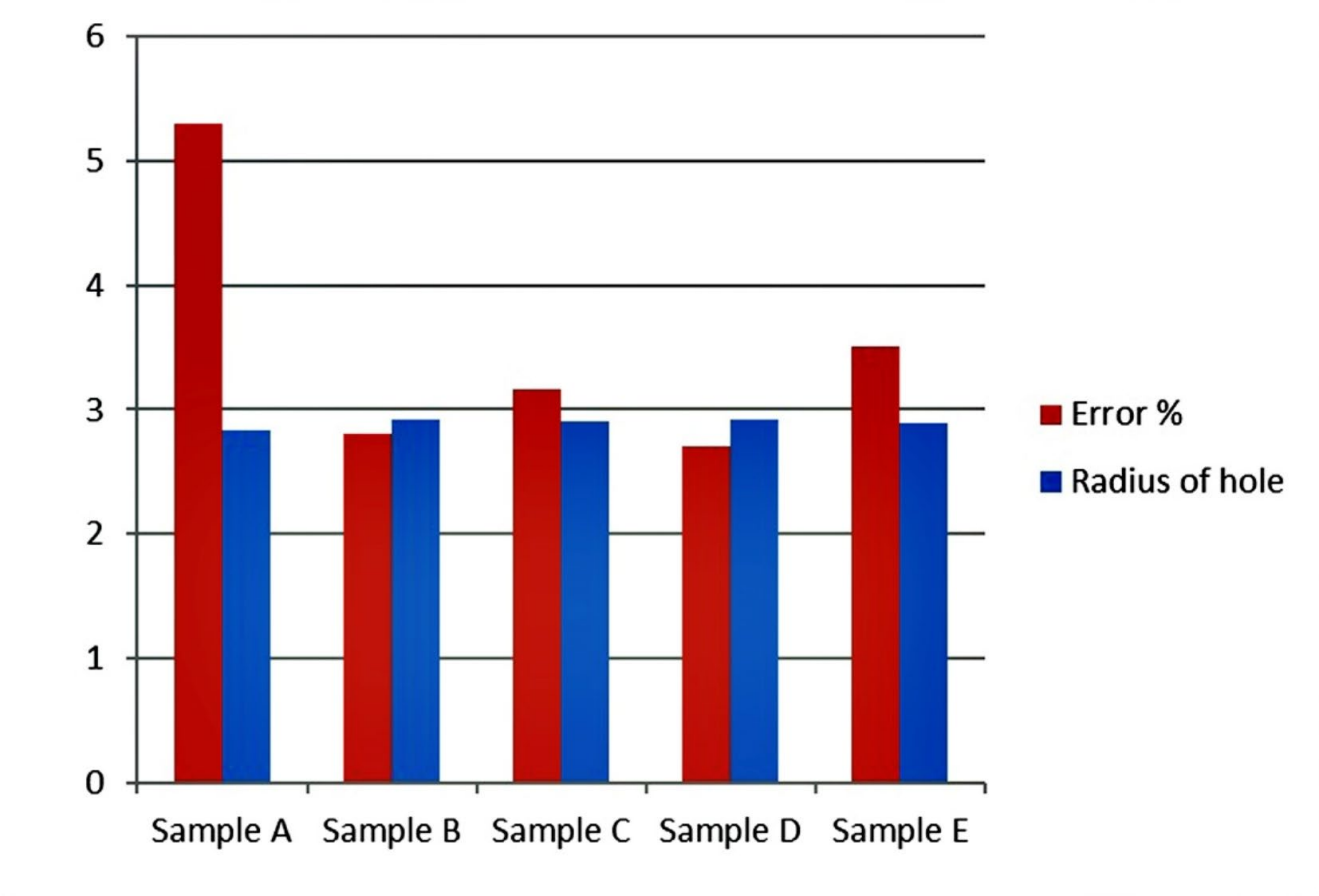
Graph showing error percentage and radius of hole in machinabilty test. *Here, Radius of Hole is in mm and the error percentage is in %.

It is seen that sample A Kenaf fiber composite shows an error percentage of 5.3%, which is more when compared to other composites. The addition of graphene and Glass fiber in natural fiber composites shows improvement in the drilling efficiency of the composites (Fig. 20).

### Fourier transform infrared spectroscopy (FTIR)

FTIR analysis is an analysis tool in which we distinguish materials. This process uses infrared light to scan specimens and determines its chemical properties. The infrared radiation of around 10,000 to 100 cm-1 through the specimen, some radiation is absorbed in the specimen, and some radiation is passed through it. The assimilated radiation is changed over into rotational or vibrational vitality. This Rotational/ Vibration energy is now converted into Spectrum through an interferogram. Every material has a unique molecular footprint, which makes the FTIR examination a magnificent tool for analyzing materials^27,28^.

FTIR analysis:

- Determines unknown materials (e.g., solids, liquids, particles)
- Checks whether there is contamination in the material (e.g., particles, films, powders)
- Distinguishes added substances after extraction from a polymer lattice.

FTIR analysis was carried out for four specimens. The results are present below.

**Fig. 21:**
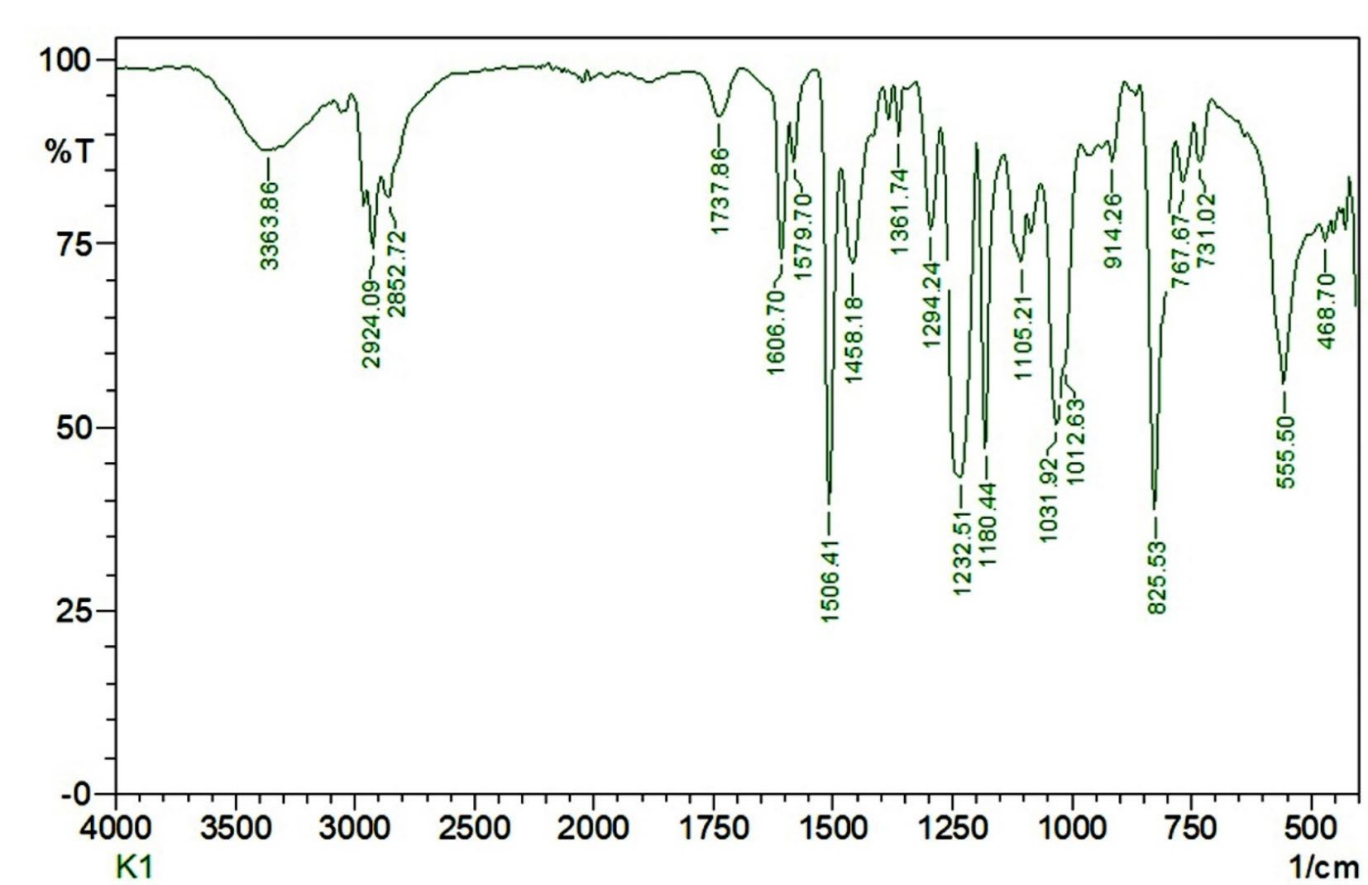
FTIR Spectrum of 0.5% Graphene (by wt) Reinforced Kenaf Fiber Composites.

The peak ranges from 3363.86 to 3365.78 cm^-1^ shows (Fig. 21) the blending of Kenaf fiber with the graphene reinforced Epoxy LY 551 matrix. The inclusion of graphene with the kenaf led to the expansion of the bands at this span additionally than the IR peak at this ambit from normal fibers. Utilization of microwave energy to aid the treatment of the strands exposed the cellulose to more antagonistic deposition of graphene particles because of its increased facet area, and eventually, a superior bond is formed between the graphene-epoxy & kenaf. Kenaf fiber composites usually show broadband in the span of 2800 to 2900 cm^-1^, which also indicates the C-H absorption band. The bands at 2924 cm^-1^ show that there are CH3 groups present in the NFC, which could be a credit to the low molecular weight of graphene in the composites. The C–H stretching vibrations, C–H bending vibrations, and C–O–C stretching vibrations are characterized by 2924.09 cm^-1^, 1458.18 cm^-1^, and 1294.24 cm^-1^ respectively. The transition bands at 1031.92 cm^-1^, 1105.21 cm^-1^, and 1232.51 cm^-1^ show the symmetric, asymmetric, and bending vibrations, respectively, of CH3 and C–O–C groups.

**Fig. 22:**
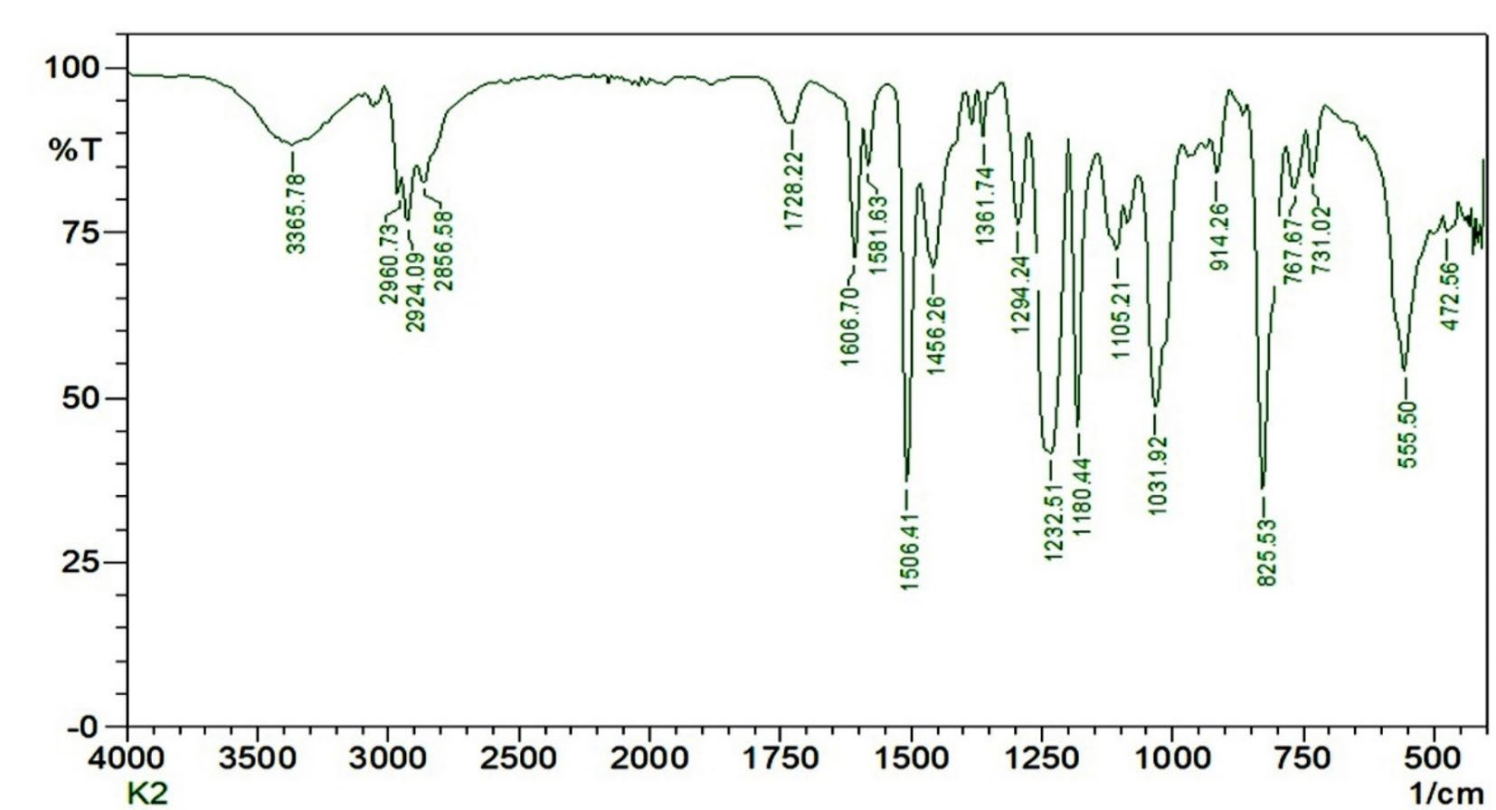
FTIR Spectrum of 1.0% Graphene (by wt) Reinforced Kenaf Fiber Composites.

The FTIR analysis resulted (Fig. 22) in the Alkali treated kenaf fiber composites; the peaks do not disappear at 600 cm^-1^ and 1850 cm^-1^, which confirmed the incomplete removal of cellulose content even after the mercerization of the kenaf fibers. The FTIR Spectroscopy showed the presence of cellulose content in the kenaf and flax fiber and presence of CH3 groups, C-H and C–O–C bands.

Composites of all two graphene compositions are used in this research and the result shows that there is an absence of a carbonyl group. This is a functional group which has a carbon double bonded to an oxygen atom C = O. This checks the removal of hemicellulose in the natural kenaf fibre. Therefore, the attainment of alkali treatment is confirmed.

Previous literature have confirmed that the band shows the existence of an aromatic carbon double bond. Further, the bands shows the existence of C–O extending bonds. The peaks confirmed the C–H alkane extending bonds. C–H bonds which are identified by the bands confirms the presence of C–C bond and aromatic nitro compounds. The extending of conjugate alkene bond which is also present in the epoxy and graphene is detected.

## Conclusion

Graphene reinforced kenaf composites were fabricated using the Conventional hand layup method. Few specimens also contained a layer of Glass fiber in between natural fiber layers. Tests were performed to compare them with the composites with only natural fiber layers and with each other. This research was carried out in order to compare natural fiber composites with the other material composites and also to compare their mechanical properties as well as water absorption properties. We have incorporated the minimum possible use of graphene in our composite optimum result attained. Natural fiber composites are environment friendly as well as inexpensive from other composites, but in our experiments, it showed similar properties when compared to its expensive.

The main objective of inclusion of graphene is to improve the mechanical properties of hybrid composites. Graphene is very significant material used for reinforcement because of its excellent electrical, mechanical and thermal properties. It has a thickness of a single atom and a two dimensional honeycomb structure. It has now achieved to be an extraordinary filler for epoxy resin composites as it provides excellent strength. The sole reason for this is the availability of the oxide group in graphene powder, which acts as a strong binder in the matrix.

The fibre matrix adhesion can be enhanced with the help of coupling agents called as epoxy. This warrants that there is a strong bond between the fibres and the matrix as it has possess very good adhesive properties.

Glass Fiber incorporated composite with 1.0% graphene reinforced has the best flexural strength. After performing the test and critically analyzing the results obtained from our experiment with other research papers, we can conclude that our specimens’ consumed less water because of the occupancy of graphene particles in the matrix. Overall, the incorporation of graphene in natural fiber composites leads to an increase in its mechanical properties. With the incorporation of graphene and glass fiber, the impact strength increases for Kenaf fiber composites. FTIR test was conducted to conclude the interferential adhesion and homogeneous distribution of fibres in the composite matrix.

### Tensile test

Tensile testing was conducted on the samples carried out on Digital tensile testing machine TFT-10KN C on ASTM D3039 specimens and the following results were obtained. The value measured of tensile strength is highest in the case of Kenaf-Glass fiber composite reinforced with 1.0% Graphene, which is around 20.3 MPa and of Kenaf Fiber with 1.0% Graphene, which is 17.5 MPa. The Force position graph shows a linear and uniform relationship between position and force over the application of a quasi-static load rate of 1 mm/min. The incorporation of glass fiber in Natural Fiber composites shows better tensile strength than plain fiber composites. The Ratio of Fiber: Epoxy in the Matrix is 25:75 (by Weight %). From the graph, it is evident that maximum load values for Kenaf-epoxy-Glass Fiber 1.0% graphene reinforced composite is 1520 N and for Kenaf-epoxy 1.0% graphene reinforced composite is 1310 N, respectively. Hence the former has higher tensile strength. There is a similar pattern observed in Flax composites in which the glass fiber incorporation increases the tensile strength. The better adhesion in the matrix leads to a better reinforcement effect. More is the amount of epoxy; better is the adhesion effect. This leads to a desirable distribution and allocation of the natural fibers in the matrix and the voids between the fibers are also bridged effectively. The stress distribution is favorable because of the presence of glass fiber which results in better strength.

### Flexural test

The three-point bending test or flexural test was done on UTM using INSTRON 8801. Tests were set up as per the ASTM D790 standard, and the following results were obtained. The variations in the stress–strain relationship indicate the change in flexural strength of the composites on glass fiber incorporation and change in the composition of graphene in the holding matrix. From the results, graphene reinforced Kenaf fiber composite has preferred flexural quality on account of the fine structure of the kenaf and subsequently better support impact. Further, for each type of fiber composites, Glass Fiber incorporated composite with 1.0% graphene reinforced has the best flexural strength. Kenaf-epoxy-Glassfiber-1.0% GKFC has the most flexural strength value of 114 N.

### Impact test

Impact test or Charpy test was performed on Impact Testing Machine using FIT 300D for the specimens made as per the ASTM D256 standard, and the following results were obtained. With the incorporation of Graphene and Glass fiber, the impact strength increases for Kenaf fiber composites. The Impact Strength was maximum for Kenaf Fiber-Epoxy-Glassfiber-0.5% graphene composite which is 3.20 N and impacts the energy of 0.05 N/mm^2^. Hence, it is suggested that the consolidation of Graphene and Glass fiber in the matrix can increase the impact strength of the composite material.

### Water absorption test

The water absorption test is done on our specimen according to ASTM standards, and the following results were obtained. The factors that influence water absorption tests are the type of specimen sample, temperature, and the materials used. After performing the test and critically analyzing the results obtained from our experiment with other research papers, we can conclude that our specimens’ consumed less water because of the occupancy of graphene particles in the matrix. It is observed that the specimen with a combination of Graphene and a layer of glass Fiber gives the optimum result for Kenaf fiber composites. It showed that Graphene reinforced NFC’s have good retention against water and implemented in automotive exteriors, which are quite exposed to the atmosphere.

### Fourier Transform infrared spectroscopy

FTIR analysis showed the presence of various organic groups, facet binding between the fiber and the matrix, etc. Overall, results shows that the incorporation of Graphene in Natural fiber composites leads to an increase in its mechanical properties. Hybrid composites that are produced by mixing natural fibers give good mechanical properties. Each specimen shows excellent and satisfying results, but the samples in which glass fiber is incorporated with natural fibers, show the optimum result in most of the tests performed.

Further, FTIR study was done which confirmed the existence of various functional groups in the composite specimens. The organic solvents were identified which showed that there are different extending trends within the bond of the molecule. This is due to the accessibility of different chemical structures.

This investigation is a base for future researchers who will use the same set of materials such as coir, graphene and e-glass fibre. The kenaf composites used in this study finds their use in potential applications such as defence, biomedical services, automobiles, packaging, and actuators, etc. Further, these will be useful for the engineers, whenever there is a demand for fabricating lightweight and high strength composite material.

## Data Availability

The datasets generated and/or analysed during the current study are not publicly available due to institution norms but are available from the corresponding author on reasonable request.
